# Myeloid *Mir34a* suppresses colitis-associated colon cancer: characterization of mediators by single-cell RNA sequencing

**DOI:** 10.1038/s41418-024-01380-9

**Published:** 2024-10-18

**Authors:** Janine König, Matjaz Rokavec, Meryem Gülfem Öner-Ziegler, Ye Fei, Heiko Hermeking

**Affiliations:** 1 Experimental and Molecular Pathology, Institute of Pathology, Faculty of Medicine, Ludwig-Maximilians-Universität München, Thalkirchner Str. 36, D-80337 Munich, Germany; 2 German Cancer Consortium (DKTK), Partner site Munich, D-80336 Munich, Germany; 3 German Cancer Research Center (DKFZ), D-69120 Heidelberg, Germany

**Keywords:** Cancer microenvironment, Experimental models of disease

## Abstract

We have previously shown that general deletion of the gene encoding the p53-inducible Mir34a microRNA enhances the number and invasion of colitis-associated colorectal cancers (CACs) in mice. Since the p53-pathway has been implicated in tumor-suppression mediated by cells in the tumor microenvironment (TME) we deleted *Mir34a* in myeloid cells and characterized CACs in these with scRNA-Seq (single cell RNA sequencing). This revealed an increase in specific macrophage subtypes, such as *Cdk8*^+^ macrophages and *Mrc1*^+^, M2-like macrophages. The latter displayed elevated expression of 21 known Mir34a target mRNAs, including *Csf1r, Axl, Foxp1, Ccr1, Nampt, and Tgfbr2*, and 32 predicted Mir34a target mRNAs. Furthermore, *Mir34a*-deficient BMDMs showed enhanced migration, elevated expression of *Csf1r* and a shift towards M2-like polarization when compared to *Mir34a*-proficient BMDMs. Concomitant deletion of *Csf1r* or treatment with a *Csf1r* inhibitor reduced the CAC burden and invasion in these mice. Notably, loss of myeloid Mir34a function resulted in a prominent, inflammatory CAC cell subtype, which displayed epithelial and macrophage markers. These cells displayed high levels of the EMT transcription factor Zeb2 and may therefore enhance the invasiveness of CACs. Taken together, our results provide in vivo evidence for a tumor suppressive role of myeloid *Mir34a* in CACs which is, at least in part, mediated by maintaining macrophages in an M1-like state via repression of Mir34a targets, such as *Csf1r*. Collectively, these findings may serve to identify new therapeutic targets and approaches for treatment of CAC.

## Introduction

Chronic inflammation of the intestine, as observed in ulcerative colitis (UC) and inflammatory bowel disease (IBD), often results in colitis-associated colorectal cancers (CAC; [1]). Patients with CACs have a poorer prognosis than patients with sporadic or inherited colorectal cancers (CRCs; [1–4]). The genetic alterations that contribute to sporadic CRC and CACs are similar but occur in a different order [2]. For example, the mutational inactivation of *p53* is an early event in CAC, whereas it occurs late in sporadic CRC [2].

Among the p53-induced microRNAs (miRNAs), miR-34a stands out as the most strongly induced miRNA according to numerous comprehensive expression studies [5]. We had previously observed that germline deletion of *Mir34a* enhances tumor initiation, progression, and invasion in a murine model of colitis-associated colon cancer [6]. However, it remained unknown whether the effects of the *Mir34a* inactivation were tumor cell autonomous, or whether *Mir34a*-deficient cells within the tumor stroma were also involved.

The tumor microenvironment plays an important role in tumor progression [7], as it contributes to invasion and therapy resistance by modulating cell-cell interactions and altering cytokine and chemokine signaling (reviewed in ref. [8]). Macrophages are an important part of the tumor microenvironment. It has been shown ex vivo that miR-34a inhibits polarization towards M2-macrophages [9, 10], which are typically alternatively activated, tumor-associated macrophages (TAM) and are known to enhance tumor growth, angiogenesis, invasion, metastasis and therapy resistance [11–13]. Furthermore, M2-polarization of macrophages is promoted by growth factors, such as the colony-stimulating factor (CSF-1), and cytokines, like IL-4, IL-13 and IL-10, released by cells in the tumor-microenvironment (reviewed in ref. [11]).

Colony-stimulating factor 1 receptor (CSF1R) is the receptor for CSF1, also known as macrophage colony-stimulating factor (M-CSF), which, among other functions, is involved in the differentiation of hematopoietic stem cells into macrophages [14–16] and is secreted by epithelial tumor cells after *p53* inactivation [17]. Recently, we showed that *CSF1R* is a direct, conserved target of *miR-34a* in human CRC cells [18] and in a murine model of inherited CRC [19]. Deletion of *Mir34a* in *Apc*^*min/+*^ mice resulted in increased adenoma formation, increased immune cell infiltration and decreased survival of mice. Concomitant intestinal-epithelial-cell (IEC) specific deletion of *Csf1r* reversed these effects [19]. Targeting CSF1R appears to be a promising anti-tumor strategy [11] and the presence of CSF1R-positive macrophages is associated with poor survival [20, 21].

Here, we determined that myeloid cell-specific deletion of *Mir34a* resulted in more invasive CACs, which displayed increased infiltration by macrophages and neutrophils. A comprehensive characterization of CACs and their tumor microenvironment (TME) by scRNA-Seq identified an increase of specific macrophage subtypes in *Mir34a*^*ΔMye*^ CACs as well as elevated levels of *Csf1r* expression in *Mir34a*^*ΔMye*^ macrophages. Deletion or therapeutic inhibition of *Csf1r* reduced the tumor burden in these mice. In addition, numerous *Mir34a* mRNA targets were significantly upregulated in *Mir34a*-deficient macrophages and neutrophils. *Mir34a*-deficient bone-marrow derived macrophages (BMDMs) showed increased migration, elevated levels of Csf1r as well as upregulation of M2 markers whereas the expression levels of M1 markers decreased. Finally, scRNA-Seq revealed a prominent population of inflammatory tumor cells with high expression of chemokines and cytokines as well as macrophage markers in *Mir34a*^*ΔMye*^ CACs.

## Materials and methods

### Breeding and handling of mice

The generation of *Mir34a*^*fl/fl*^ mice was previously described [22]. *LysM-Cre* (*B6.129P2-Lyz*^*tm1(cre)Ifo/J*^) mice and *Csf1r*^*fl/fl*^ (*B6.Cg-Csf1r*^*tm1Jwp/J*^) were purchased from Jackson Laboratories (Stock: 004781 and Stock: 021212) [23, 24]. To delete *Mir34a* in myeloid cells (*Mir34a*^*ΔMye*^), *Mir34a*^*fl/fl*^ mice were crossed with *LysM-Cre* mice. *Mir34a*^*ΔMye*^ mice were crossed with *Csf1r*^*fl/fl*^ mice to obtain *Mir34a; Csf1r*^*ΔMye*^ mice. For generation of *Csf1r*^*ΔMye*^ mice, *LysM-Cre* mice were crossed with *Csf1r*^*fl/fl*^ mice. All mice were backcrossed on C57Bl/6 background for at least five generations. Co-housed *Mir34a*-proficient *Mir34a*^*fl/fl*^ littermates were used as controls in all experiments. Mice at 6–10 weeks of age were intraperitoneally (i.p.) injected with azoxymethane (AOM, 10 mg/kg) 5 days prior to the first and second dextran sodium sulfate (DSS) induced colitis period. The first and second colitis period were induced by giving 2.5% DSS dissolved in drinking water for a period of 5 days, followed by 16 days of regular water. Subsequently, the third colitis period was induced by giving 2% DSS for 5 days. All mice were sacrificed 110 days after the first AOM injection. For experiments with additional conditional deletion of *Csf1r* and inhibitor treatment, the dosage of DSS was reduced in second cycle to 2% and mice were sacrificed 100 days after the first injection. The *Csf1r* inhibitor GW2580 (Sigma-Aldrich) was given in chow (1%) with *ad libidum* access from day 60 onwards. GW2580-supplemented food was prepared by Sniff Special Diets (Soest, Germany). Mice were kept on a 12 h light/dark cycle in individually ventilated cages in a specific pathogen-free facility at Institute of Pathology, Ludwig-Maximilians-University Munich, with chow (standard formulation) and water supply *ad libitum*. All animal protocols were approved by the local authorities (Regierung von Oberbayern, AZ: 55.2-1-54-2532-201-2014; AZ:55.2-2532.Vet_02-21-111). Genotyping primers are listed in Table S3.

### Single cell isolation from murine CACs

6–8 CACs from 3 female mice per genotype were isolated and dissociated according to the 10× Genomics protocol “Tumor Dissociation for Single Cell RNA Sequencing” (CG000147 Rev B). In brief, isolated CACs were lysed using the Mouse Tumor Dissociation kit in gentleMACS C tubes using the gentleMACS Octo Dissociator with heaters (Miltenyi Biotec). The lysis of red blood cells was performed using 1× Red Blood Cell Lysis Solution (Miltenyi Biotec). To increase the percentage of viable cells the protocol “Removal of Dead Cells for Single Cell RNA Sequencing” (10x Genomics, CG000093 Rev C) was employed according to manufacturer’s instructions. For this, the MACS Dead Cell Removal kit and MS Columns with the OctoMACS separator on the MACS multistand (Miltenyi Biotec) were used.

### scRNA-Seq analysis

Single cells were loaded on the 10× Chromium system and labeled with the 3′-CellPlex kit set A (10× Genomics) according to the manufacturer’s recommendations. For each genotype 30,000 cells were loaded. Barcoded sequencing libraries were prepared with the Chromium Single Cell 3′ v.3.1 Gene Expression Kit (10× Genomics). Paired-end sequencing (2 × 100 bp) was performed on Illumina NovaSeq 6000 (Illumina) to obtain 90,000 reads/cell using the standard workflow.

### scRNA-Seq data analysis

Cellranger v7.2.0 software (10× Genomics) was used to align reads to the refdata-gex-mm10-2020-A mouse reference transcriptome and generate a gene-cell unique molecular identifier count matrix for each cell. Cells with more than 200 expressed mRNAs and less than 25% mitochondrial RNA content were selected for further analysis. Moreover, cell doublets were excluded by applying the Scrublet package [25]. The gene expression matrices were normalized using the LogNormalize function of Seurat v5.01 [26]. Next, the Seurat FindVariableFeatures, FindIntegrationAnchors, and IntegrateData functions were used to obtain 2000 of the most variant mRNAs and perform integration with batch correction. Cell clustering was performed by using the Seurat RunPCA, RunUMAP, and Findclusters functions with resolutions from 0.5 to 1.5 for best cluster representation. Cell-type-specific mRNAs and differentially expressed mRNAs between genotypes were identified by the Seurat function FindMarkers. Gene set enrichment analyses were performed using clusterProfiler [27]. The significance of differences in the abundance of cell types/clusters in CACs derived from *Mir34a*^*ΔMye*^ vs. *Mir34a*^*F/F*^ mice was calculated using a single-cell specific method based on the mixed-effect modeling of associations on single cells (MASC) [28]. Detailed results are provided in Table S4.

### Analysis of intercellular ligand-receptor communications

Intercellular communication was analyzed using CellChat [29]. Clustered UMAPs generated by Seurat were imported to CellChat and converted to CellChat objects. Results are presented as Circo plots using the netVisual_aggregate CellChat function.

### Cell differentiation trajectory analysis

Cell differentiation trajectories were estimated based on pseudotime calculated by three different algorithms: Palantir [30], Monocle3 [31], and SlingShot [32]. The naive state of lineages was used as the root node/early cell. The trajectories are shown on the UMAPs calculated with Seurat as described above. To analyze RNA velocity, velocyto [33] was first used to separate unspliced and spliced mRNAs in each cell. Next, scVelo [34] was used to calculate RNA velocity vectors. To determine cell lineages, initial and terminal macrostates were determined using CellRank [35]. For the visualization of gene expression trends for each lineage the expression data was imputed with MAGIC (Markov Affinity-based Graph Imputation of Cells).

### Bioinformatics analysis of public datasets

Expression and clinical data of the TCGA colon adenocarcinoma (COAD) and rectal adenocarcinoma (READ) cohorts was obtained from the MD Anderson standardized data browser (http://bioinformatics.mdanderson.org/TCGA/databrowser/). The RNA-Seq by Expectation-Maximization (RSEM) normalized expression values from the Illumina RNASeqV2 (genes) datasets were used. Expression and clinical data of other CRC patient datasets was downloaded from NCBI GEO (www.ncbi.nlm.nih.gov/geo). The statistics for survival analysis was calculated by log-rank test. For binary classification of cases (high/low expression), the Survminer R-package (https://CRAN.R-project.org/package=survminer) was used to determine optimal cut-off values. The CMS classification of public datasets was obtained from Guiney et al. [36].

### Statistical analysis

GraphPad Prism 10.1.0 was used to determine significant differences between groups via two-tailed unpaired Students *t*-test. Values are represented as mean ± SEM. *P*-values < 0.05 were regarded as statistically significant (*p* < 0.05*; *p* < 0.01**; *p* < 0.001*** and *p* < 0.0001****).

## Results

**Fig. 1:**
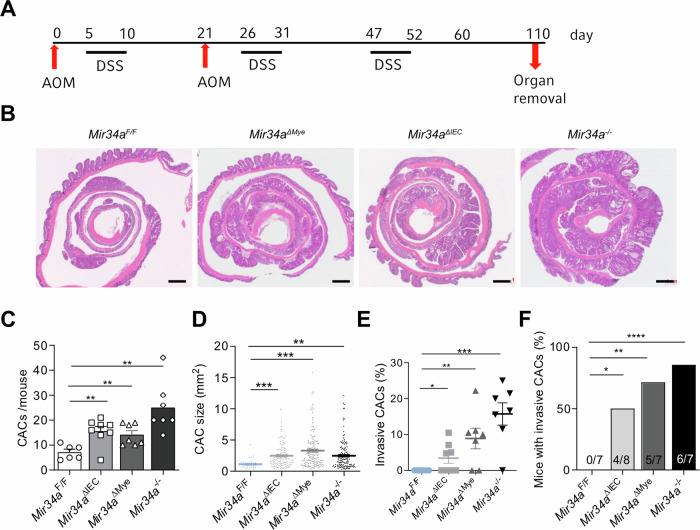
Effects of epithelial- and myeloid-specific deletion of *Mir34a* in a CAC mouse model. **A** Treatment scheme of the CAC model induced by AOM/DSS. **B** Histology of AOM/DSS challenged mice with indicated genotypes, scale bars are 1 mm. **C** CAC incidence in *Mir34a*^*F/F*^, *Mir34a*^*ΔIEC*^, *Mir34a*^*ΔMye*^ and *Mir34a*^*–/–*^ mice (*n* ≥ 7 mice per group). **D** CAC size of mice with indicated genotypes, CAC areas measured by computerized image analysis (*n* ≥ 7 mice per group). **E** Percentage of invasive CACs of indicated genotypes. **F** Number of mice with invasive CACs/total number of mice of indicated genotype. (**C**–**F**) Students *t*-test was used to determine significance. Values represent the mean ± SEM with *p* < 0.05*; *p* < 0.01**; *p* < 0.001*** and *p* < 0.0001****.

We had previously reported that germline deletion of *Mir34a* enhances tumor initiation, progression and invasion in a murine model of colitis-associated colon cancer [6]. However, it remained unknown whether the effects of the *Mir34a* inactivation were tumor cell autonomous, or whether *Mir34a*-deficient cells within the tumor stroma were also involved. In a mouse model of intestinal tumorigenesis, it was observed that p53 is a critical regulator of tumorigenesis during inflammation in macrophages [37]. Furthermore, it was shown that p53 is activated during inflammation [38] and may have non-tumor-cell-autonomous tumor suppressive functions [39]. Since *Mir34a* is directly induced by p53, it may mediate these effects of p53. To determine, which cell type mediates the effects of *Mir34a* inactivation on CAC formation, we generated *Villin-Cre/Mir34a*^*F/F*^ and *LysM-Cre/Mir34a*^*F/F*^ mice, to obtain mice with intestinal epithelial cell-specific or myeloid lineage-specific deletion of *Mir34a*, designated as *Mir34a*^*ΔIEC*^ and *Mir34a*^*ΔMye*^ mice, respectively. When CACs were induced as described in Fig. 1A, *Mir34a*^*ΔIEC*^ and *Mir34a*^*ΔMye*^ mice formed more and larger CACs than *Mir34a*^*F/F*^ mice (Fig. 1B–D). The tumor load of *Mir34a*^*ΔIEC*^ and *Mir34a*^*ΔMye*^ mice was less than in *Mir34a*^*–/–*^ mice (Fig. 1C). *Mir34a*^*ΔIEC*^, *Mir34a*^*ΔMye*^ and *Mir34a*^*–/–*^ mice displayed invasive CACs, whereas CACs in *Mir34a*^*F/F*^ mice were non-invasive (Fig. 1E). However, *Mir34a*^*ΔIEC*^ and *Mir34a*^*ΔMye*^ mice displayed less invasive CRCs than *Mir34a*^*–/–*^ mice (Fig. 1E, F). Surprisingly, tumor invasiveness was increased to a larger extent by inactivation of *Mir34a* in myeloid cells than by its deletion in intestinal epithelial cells. Taken together, these results suggested a tumor suppressive role of *Mir34a* in both, the intestinal epithelial cells and in TAMs. As the CAC-promoting effect of myeloid-specific inactivation of *Mir34a* was unexpected, we decided to characterize it in more detail.

### scRNA-Seq analysis of CACs in *Mir34a*^*ΔMye*^ mice

**Fig. 2:**
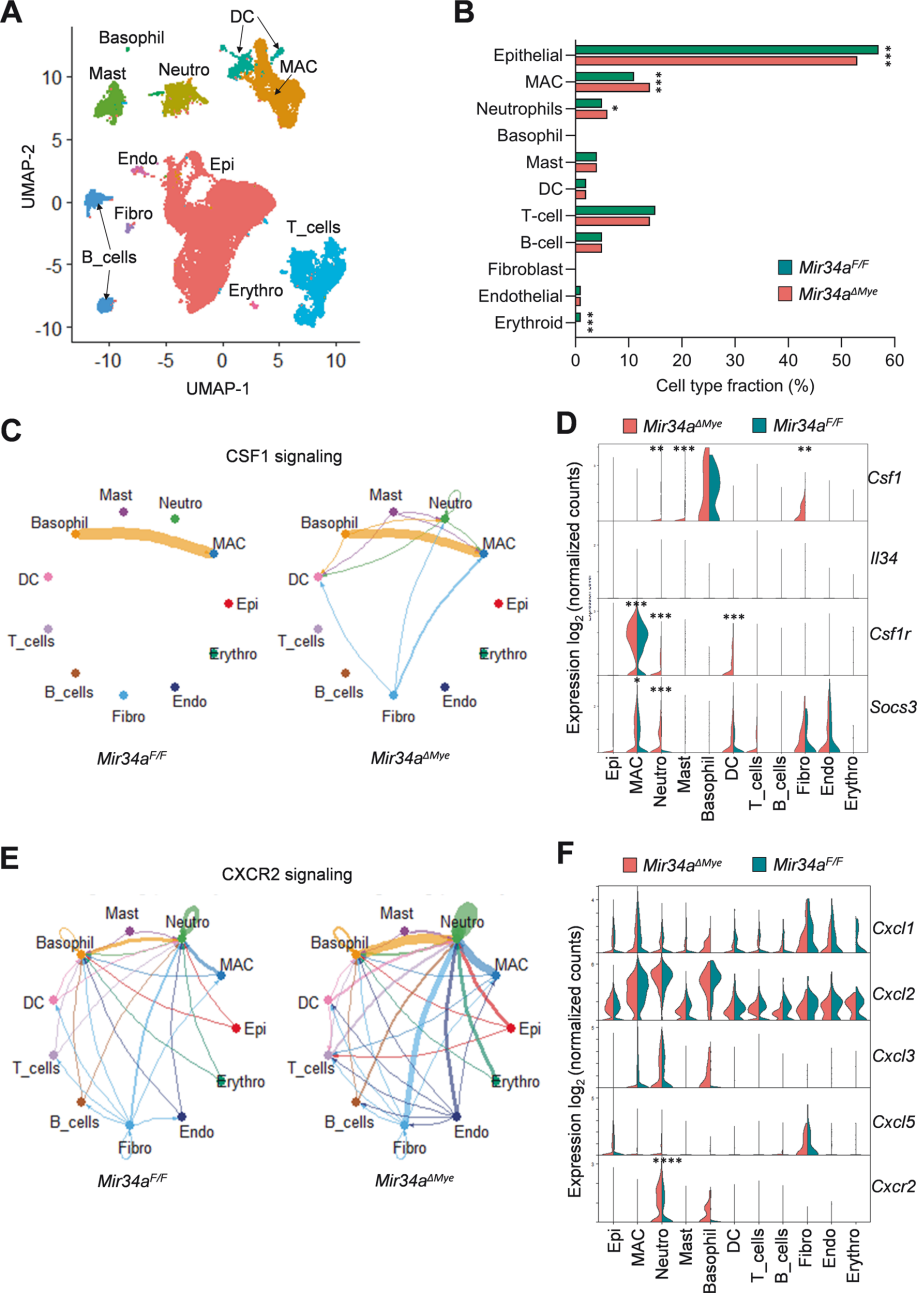
scRNA-Seq analysis of CACs and their microenvironment in mice with myeloid-specific deletion of *Mir34a.* **A** Combined UMAP plot showing subtypes of all cells (38,883 cells) from CACs, derived from *Mir34a*^*ΔMye*^ and *Mir34a*^*F/F*^ mice. **B** Proportions of major cell types in *Mir34a*^*ΔMye*^ and *Mir34a*^*F/F*^ CACs. **C** CSF1 signaling networks between indicated cell types in *Mir34a*^*ΔMye*^ and *Mir34a*^*F/F*^ CACs. The edge width represents the communication probability. **D** Expression of CSF1 signaling components in indicated cell types in *Mir34a*^*ΔMye*^ and *Mir34a*^*F/F*^ CACs. **E** CXCR2 signaling networks between indicated cell types in *Mir34a*^*ΔMye*^ and *Mir34a*^*F/F*^ CACs. The edge width represents the communication probability. **F** Expression of CXCR2 signaling components in indicated cell types in *Mir34a*^*ΔMye*^ and *Mir34a*^*F/F*^ CACs. Significance was determined as described in the methods section scRNA-Seq data analysis (*p* < 0.05*; *p* < 0.01**; *p* < 0.001*** and *p* < 0.0001****). The mRNAs not marked with asterices did not reach significance in their differential expression.

To comprehensively characterize cell types and differential gene expression in CACs and their TME after myeloid-specific *Mir34a*-inactivation, we performed single-cell RNA sequencing (scRNA-Seq) analysis of CACs derived from *Mir34a*^*ΔMye*^ mice and *Mir34a*^*F/F*^ mice. We isolated 6–8 tumors from three mice for each genotype and combined these for scRNA-Seq analyses. After quality control, 38,883 single-cell transcriptomes were obtained (18,571 from *Mir34a*^*ΔMye*^ CACs and 20,312 from *Mir34a*^*F/F*^ CACs). Cell-types were clustered based on the scRNA-Seq results using a uniform manifold approximation and projection (UMAP) plot (Fig. 2A). By utilizing cell-type-specific marker-based annotations, eleven major cell-type subtypes were identified (Fig. 2A and Supplementary Fig. S1A, B). CACs from *Mir34a*^*ΔMye*^ mice showed an increase of macrophages and neutrophils, and a decrease of tumor epithelial cells when compared to *Mir34a*^*F/F*^ CACs (Fig. 2B). As determined by Cellchat, Csf1/Csf1r signaling, which plays an important role in macrophage recruitment and polarization [40], was elevated in *Mir34a*^*ΔMye*^ CACs (Fig. 2C). This increase is presumably based on the elevated expression of the *Csf1* ligand in neutrophils, mast cells, fibroblasts and basophils as well as on the elevated levels of *Csf1r* in macrophages, neutrophils, and dendritic cells in *Mir34a*^*ΔMye*^ CACs (Fig. 2D). Moreover, *Mir34a*^*ΔMye*^ macrophages displayed elevated expression of *Socs3*, which is a target of Stat3 signaling that is activated by Csf1r [40] (Fig. 2D). Furthermore, Cellchat analyses showed that Cxcr2 signaling, which plays an important role in neutrophil recruitment [41, 42] was elevated in *Mir34a*^*ΔMye*^ CACs (Fig. 2E), which may be due to the elevated expression of *Cxcr2* receptor in neutrophils and increased expression of *Cxcl1* and *Cxcl3* in basophils (Fig. 2F). Moreover, *Mir34a*^*ΔMye*^ neutrophils show elevated expression of *Socs3* (Fig. 2D), which can be induced by Cxcr2-mediated activation of Stat3 [43]. These results imply that the elevated Csf1r and Cxcr2 signaling observed in *Mir34a*^*ΔMye*^ CACs could be responsible for a stronger recruitment and a consequent increase of macrophages and neutrophils.

### scRNA-Seq analysis of CAC cells

**Fig. 3:**
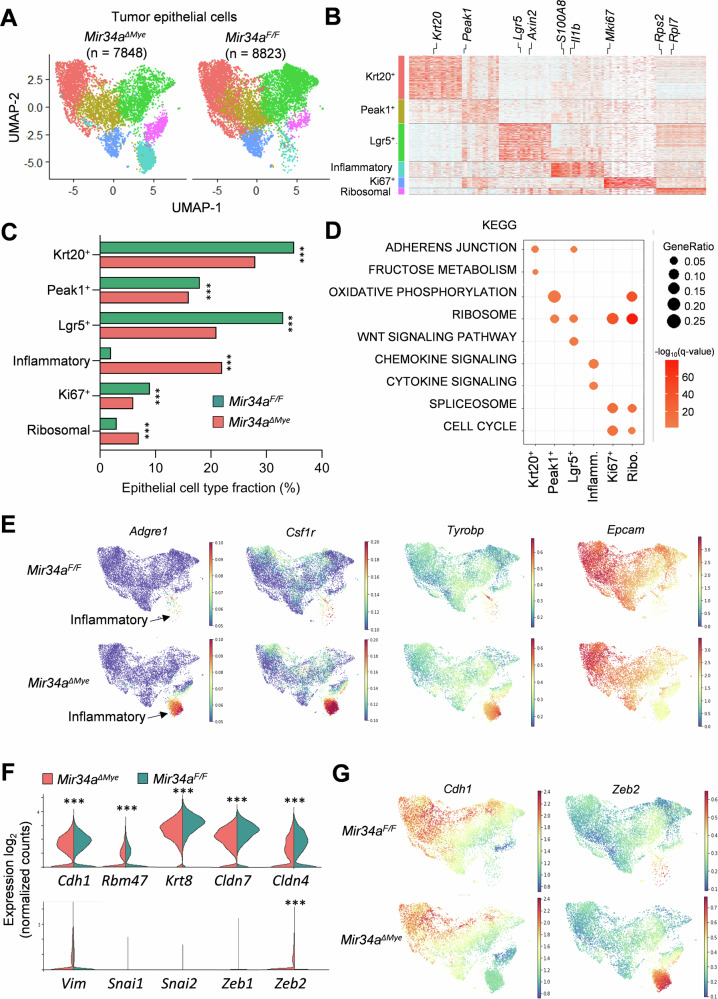
scRNA-Seq analysis of CAC cells in mice with myeloid-specific deletion of *Mir34a.* **A** UMAP plot showing subtypes of tumor epithelial cells from CACs, derived from *Mir34a*^*ΔMye*^ and *Mir34a*^*F/F*^ mice. **B** Heatmap of top ten differentially expressed genes in each tumor epithelial cell subtype. **C** Proportions of tumor epithelial cell subtypes in *Mir34a*^*ΔMye*^ and *Mir34a*^*F/F*^ CACs. **D** Enrichment of indicated MsigDB Hallmark gene sets in differentially expressed genes for each tumor epithelial cell subtype. **E** UMAP plots of tumor epithelial cells colored by the expression of indicated mRNAs. **F** Expression of indicated EMT-related mRNAs in *Mir34a*^*ΔMye*^ and *Mir34a*^*F/F*^ tumor epithelial cells. **G** UMAP plots of tumor epithelial cells colored by the expression of indicated EMT-related mRNAs. Significance was determined as described in the methods section scRNA-Seq data analysis (*p* < 0.001***). The mRNAs not marked with asterices did not reach significance in their differential expression.

Next, we divided CAC cells (7848 cells from *Mir34a*^*ΔMye*^ CACs and 8823 cells from *Mir34a*^*F/F*^ CACs) into six subtypes (Krt20+, Peak1+, Lgr5+, Inflammatory, Ki67+, and Ribosomal) (Fig. 3A, B). These included a subtype characterized by high expression of the colonic stem cell marker *Lgr5* and elevated Wnt signaling, a subtype with high levels of the pan-differentiation marker *Krt20*, and a subtype of proliferating cells, which were positive for *Ki67* (Fig. 3B). These 3 subtypes were more abundant in *Mir34a*^*F/F*^ CACs (Fig. 3C). In contrast, a subtype characterized by inflammatory chemokine and cytokine signaling was increased in *Mir34a*^*ΔMye*^ CACs (Fig. 3C, D). These cells expressed pro-inflammatory genes, such as *Cd74*, *S100a8*, and *S100a9*, which have been shown to contribute to the progression of various cancer types [44, 45] (Fig. S2). Beside epithelial markers, such as *Epcam*, these cells also expressed macrophage marker genes, including *Csf1r* and *Adgre1* (F4/80), as well as the macrophage fusion receptor *Tyrobp* (DAP12) (Fig. 3E). We also analyzed the expression of *Tyrobp* mRNA in the publicly available RNA-Seq data (SRP097890) obtained with the iKAP mouse model of metastatic CRC. The iKAP mouse model harbors conditional alleles of the most common mutations found in human CRC allowing to recapitulate CRC progression, i.e., *K-RAS,* *APC* and *p53* [46]. The expression of *Tyrobp* was lowest in normal colon (NC), increased in non- and low-invasive tumors (TA and T1) and reached its highest level in tumors that invade through the serosa and the outer intestinal wall (T4) and was still elevated in the corresponding liver metastases (Fig. S3A). In human CRC patients, high expression of *Tyrobp* was significantly associated with poor survival in the majority of ten analyzed CRC patient cohorts (Fig. S3B). Since *Mir34a*^*ΔMye*^ CACs showed increased invasion (Fig. 1E), we analyzed the expression of EMT-associated genes, which play an important role in tumor invasion [47]. The expression of epithelial cell state-associated genes *Cdh1, Rbm47, Krt8, Cldn7*, and *Cldn4* was decreased in *Mir34a*^*ΔMye*^ CAC cells, whereas the expression of the mesenchymal cell state-associated gene *Zeb2* was increased (Fig. 3F). However, the expression of the mesenchymal cell state associated genes *Vim, Snai1, Snai2*, and *Zeb1* did not differ (Fig. 3F), suggesting that CAC cells in *Mir34a*^*ΔMye*^ tumors underwent a partial EMT. Epithelial cell state-associated genes including *Cdh1* were pre-dominantly expressed in the  more differentiated, Krt20^+^ CAC cells and decreased in the inflammatory CAC cells (Fig. 3G). In contrary, the mesenchymal cell state associated gene *Zeb2* was mainly expressed in the inflammatory CAC cells (Fig. 3G). In the iKAP mouse model the expression of *Zeb2* increased from normal colon (NC) towards non- and low-invasive tumors (TA and T1) and reached its highest level in tumors that invade through the serosa and the outer intestinal wall (T4) (Fig. S3C). In human CRC patients, high expression of Zeb2 was significantly associated with poor survival in the majority of ten analyzed CRC patient cohorts (Fig. S3D). Taken together, myeloid *Mir34a*-deficient CACs show a prominent increase of cancer cells with enhanced inflammatory signaling, which display a partially mesenchymal signature and may therefore represent cancer cells with enhanced migratory and invasive capacities.

### scRNA-Seq analysis of neutrophils in the TME

**Fig. 4:**
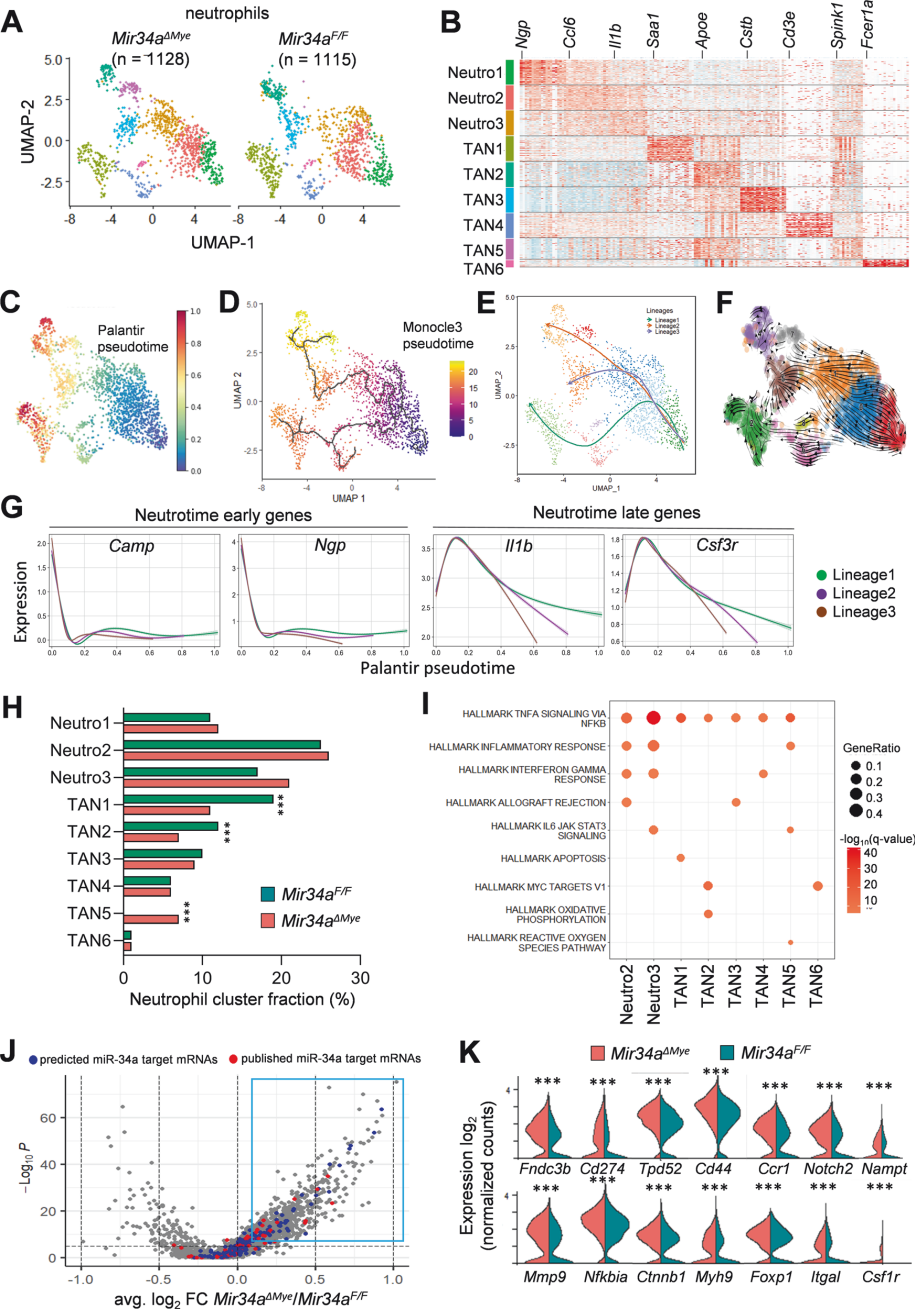
scRNA-Seq analysis of CAC-associated neutrophils in mice with myeloid-specific deletion of *Mir34a.* **A** UMAP plot showing subtypes of neutrophils in CACs derived from *Mir34a*^*ΔMye*^ and *Mir34a*^*F/F*^ mice. **B** Heatmap of the top ten differentially expressed genes in each neutrophil subtype. **C** UMAP plot showing the Palantir pseudotime of neutrophils. The color indicates pseudotime directionality from the earliest (blue) to the latest (red). **D** UMAP plot showing the Monocle analysis of neutrophils. The color indicates pseudotime directionality from the earliest (blue) to the latest (yellow). **E** UMAP plot showing the Slingshot analysis of neutrophils. Terminal cell lineages are indicated. **F** RNA velocity analysis of neutrophil subtypes with vector fields representing RNA velocity projected onto the UMAP plot. **G** Expression trends of indicated Neutrotime mRNAs for the indicated neutrophil lineages. **H** Proportions of neutrophil subtypes in *Mir34a*^*ΔMye*^ and *Mir34a*^*F/F*^ CACs. **I** Enrichment of indicated MsigDB Hallmark gene sets in differentially expressed genes for each neutrophil subtype. **J** Volcano plot of genes differentially expressed between *Mir34a*^*ΔMye*^ and *Mir34a*^*F/F*^ neutrophils. Predicted and published miR-34a targets are indicated in blue and red, respectively. **K** Expression of indicated miR-34a target mRNAs in *Mir34a*^*ΔMye*^ and *Mir34a*^*F/F*^ neutrophils. Significance was determined as described in the methods section scRNA-Seq data analysis (*p* < 0.001***). The mRNAs not marked with asterices did not reach significance in their differential expression.

Sub-clustering of neutrophils (1128 cells from *Mir34a*^*ΔMye*^ CACs and 1115 cells from *Mir34a*^*F/F*^ CACs) revealed nine subtypes (Fig. 4A, B). Differentiation trajectory and RNA velocity analyses predicted three lineages of neutrophil differentiation, which initiate at subtype Neutro1, continue via subtypes Neutro2 and Neutro3, and terminate at subtypes TAN1, TAN2, and TAN3 (Fig. 4C–F). Recently, a “neutrotime” transcriptional signature has been characterized, which includes mRNAs encoding factors relevant for differentiation of pre-neutrophils residing in the bone marrow into mature neutrophils in blood and spleen [48]. Our results show that during neutrophil differentiation in *Mir34a*^*F/F*^ and *Mir34a*^*ΔMye*^ CACs the expression of early neutrotime genes decreases, whereas the expression of late neutrotime genes initially increases and then also decreases (Fig. 4G). These results suggest that the differentiation of neutrophils from subtype Neutro1 to subtypes Neutro2 and Neutro3 recapitulates the neutrotime lineage in healthy neutrophil populations, whereas other subtypes might represent tumor associated neutrophils (TAN). The abundance of neutrophil subtypes between *Mir34a*^*F/F*^ and *Mir34a*^*ΔMye*^ mice was similar except for subtype TAN5, which was present primarily in *Mir34a*^*ΔMye*^ mice (Fig. 4H). These neutrophils showed an enrichment of IL6/STAT3 and reactive oxygen species (ROS) signaling (Fig. 4I). Finally, we determined differential mRNA expression between *Mir34a*^*F/F*^ and *Mir34a*^*ΔMye*^ neutrophils (Fig. 4J and extended data 1) and showed that 40 (14 expressed in at least 50% of the cells) published miR-34a targets, such as *Mmp9, Notch2, Nampt, Cd44, Foxp1* and *Csf1r*, were among the mRNAs upregulated in *Mir34a*^*ΔMye*^ neutrophils (Fig. 4K). Moreover, 59 significantly upregulated genes in *Mir34a*^*ΔMye*^ neutrophils represent potential, not yet published miR-34a targets, with 26 of them expressed in at least 50% of the cells (extended data 1). Among these miR-34a targets the increased levels of Matrix metalloproteinase 9 (Mmp9) might play an important role in the enhanced progression of *Mir34a*^*ΔMye*^ CACs, because it has been shown that neutrophil-secreted Mmp9 can break down collagen and remodel the ECM to promote tumor cell invasion and metastasis [49]. *Mmp9* was primarily expressed in Neutro1-3 neutrophil subtypes (Fig. S4). Taken together, *Mir34a*^*ΔMye*^ neutrophils show an increased expression of the miR-34a target *Mmp9*, which could contribute to the promotion of tumor progression and invasion. Furthermore, the TAN5 subtype, which showed enrichment in IL6/STAT3 and ROS signaling, was almost exclusively present in *Mir34a*^*ΔMye*^ mice.

### scRNA-Seq analysis of macrophages in the TME of CACs

**Fig. 5:**
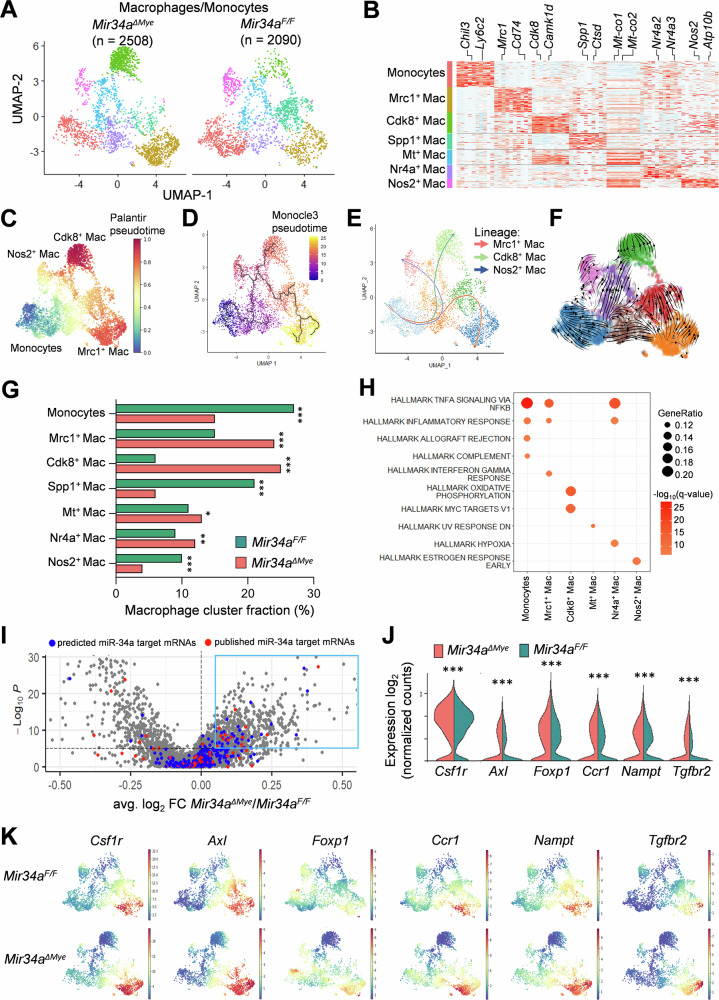
scRNA-Seq analysis of CAC-associated macrophages and monocytes in mice with myeloid-specific deletion of *Mir34a.* **A** UMAP plot showing subtypes of macrophages/monocytes from CACs, derived from *Mir34a*^*ΔMye*^ and *Mir34a*^*F/F*^ mice. **B** Heat-map of top ten differentially expressed genes in each macrophage/monocyte subtype. **C** UMAP plot showing the Palantir pseudotime of monocytes and macrophages. The color indicates pseudotime directionality from the earliest (blue) to the latest (red). **D** UMAP plot showing the Monocle analysis of monocytes and macrophages. The color indicates pseudotime directionality from the earliest (blue) to the latest (yellow). **E** UMAP plot showing the Slingshot analysis of monocytes and macrophages. Terminal cell lineages are indicated. **F** RNA velocity analysis of monocyte/macrophage subtypes with vector fields representing RNA velocity projected onto the UMAP plot. **G** Proportions of monocyte/macrophage subtypes in *Mir34a*^*ΔMye*^ and *Mir34a*^*F/F*^ CACs. **H** Enrichment of indicated MsigDB Hallmark gene sets in differentially expressed genes for each monocyte/macrophage cluster. **I** Volcano plot of differentially expressed genes between *Mir34a*^*ΔMye*^ and *Mir34a*^*F/F*^ monocyte/macrophage. Predicted and published miR-34a targets are indicated in blue and red, respectively. **J** Expression of indicated miR-34a target mRNAs in *Mir34a*^*ΔMye*^ and *Mir34a*^*F/F*^ monocytes/macrophages. **K** UMAP plots of monocytes/macrophages colored by the expression of indicated miR-34a target mRNAs. Significance was determined as described in methods section scRNA-Seq data analysis with *p* < 0.05*; *p* < 0.01** and *p* < 0.001***.

Sub-clustering analysis of macrophages and monocytes (2508 cells from *Mir34a*^*ΔMye*^ CACs and 2090 cells from *Mir34a*^*F/F*^ CACs) identified one monocyte (Ly6c2^+^) and six macrophage subtypes (Mrc1^+^, Cdk8^+^, Spp1^+^, Mitochondrial^+^, Nr4a^+^, and Nos2^+^) (Fig. 5A, B). Differentiation trajectory analyses using three algorithms (Palantir, Monocle3, and Slingshot) revealed three major lineages of macrophage differentiation: from monocytes towards Mrc1^+^, Nos2^+^, and Cdk8^+^ macrophages (Fig. 5C–E). Similar results were obtained by RNA velocity analysis, which also showed a directional flow of vector fields from monocytes towards the Mrc1^+^, Nos2^+^, and Cdk8^+^ macrophage lineages (Fig. 5F). The Spp1^+^, Mitochondrial^+^, and Nr4a^+^ subtypes presumably represent intermediate stages of macrophages. High expression of *Mrc1* is typical for M2-polarized macrophages, which exhibit pro-tumorigenic and anti-inflammatory features [50], whereas high levels of *Nos2* characterizes M1-polarized macrophages with anti-tumorigenic and pro-inflammatory characteristics [51]. The Mrc1^+^ macrophages were more abundant in *Mir34a*^*ΔMye*^ CACs (Fig. 5G), while Nos2^+^ macrophages were more abundant in *Mir34a*^*F/F*^ CACs (Fig. 5G). These results suggest that the loss of *Mir34a* shifts the polarization of macrophages towards the M2-like state. *Mir34a*^*ΔMye*^ CACs also showed a strong increase of the Cdk8^+^ macrophage subtype (Fig. 5G). *Cdk8* was shown to enhance β-catenin and MYC activity [52]. Consistently, these macrophages also expressed high levels of MYC target genes (Fig. 5H). Furthermore, Cdk8^+^ macrophages expressed elevated levels of mRNAs encoding enzymes involved in oxidative phosphorylation, which has been associated with M2 macrophage polarization [53]. Next, we determined, which mRNAs display differential expression in *Mir34a*^*F/F*^ vs. *Mir34a*^*ΔMye*^ macrophages (Fig. 5I and extended data 1). Among the mRNAs significantly upregulated in *Mir34a*^*ΔMye*^ macrophages were 21 published miR-34a targets, five of them expressed in at least 50% of the cells: *Csf1r*, *Axl*, *Foxp1*, *Ccr1*, *Nampt*, and *Tgfbr2* (Fig. 5J). The expression of these miR-34a targets was highest in Mrc1^+^ M2-like macrophages (Fig. 5K) and upregulated during differentiation into this macrophage lineage as determined by pseudotime analysis (Fig. S5A). Recently, Zhang and colleagues identified two TAM subtypes expressing *Maf* and *Mgl2* which were sensitive to anti-Csf1r treatment [54]. Here, *Maf* and *Mgl2* were predominantly expressed in the *Mrc1*^*+*^ macrophage subtype, which also expresses *Csf1r* but not *Nos2* (Fig. S5B). According to our recent miR-34a target meta-analysis [55], 32 of the significantly upregulated mRNAs in *Mir34a*^*ΔMye*^ macrophages represent potential, not yet published miR-34a targets, with seven of them expressed in at least 50% of the cells: *Btg2, Mbln1, Ell2, Prkcb, Cap1, Nf1, and Synj1* (extended data 1). The increased levels of *Csf1r* expression might play an important role in the enhanced progression of *Mir34a*^*ΔMye*^ CACs, because Csf1r plays an important role in macrophage recruitment and polarization [40]. Moreover, several other published and potential miR-34a targets that were upregulated in *Mir34a*^*ΔMye*^ macrophages, such as *Axl*, have been associated with M2-polarization and pro-tumorigenic functions in TAMs and might therefore also be involved in the progression of *Mir34a*^*ΔMye*^ CACs [56–58]. In addition, *Pdgfc*, which promotes angiogenesis (reviewed in ref. [59]) and the immunosuppressive cytokine Interleukin 10 (*Il10*), that contributes to tumor immune evasion (reviewed in ref. [60]), were upregulated in *Mir34a*^*ΔMye*^ macrophages, particularly in the Mrc1^+^ subtype (Fig. S6A, S6B). Finally, we analyzed which factors, that are secreted by macrophages, might promote tumor cell invasiveness. CellChat analyzes showed that the semaphorin signaling, which has been previously implicated in tumor progression (reviewed in ref. [61]), is elevated in *Mir34a*^*ΔMye*^ CACs (Fig. S6C). The Sema4d ligand was expressed in macrophages, neutrophils, mast cells, T-cells, and B-cells (Fig. S6D, S6E) and upregulated in *Mir34a*^*ΔMye*^ CACs (Fig. S6F). Notably, Sema4d is a potential miR-34a target according to our recent miR-34a target meta-analysis [55]. The Plxnb2, which is a receptor for Sema4d, showed the highest expression in tumor epithelial cells (Fig. S6D), suggesting that the elevated levels of Sema4d in *Mir34a*^*ΔMye*^ CACs might have an impact on tumor cells towards increased invasiveness. Taken together, these results show that myeloid *Mir34a*-deficiency leads to an increase of macrophages harboring pro-tumorigenic capacities (*Mrc1*^*+*^), presumably by upregulation of *Mir34a* target mRNAs, such as *Csf1r* and *Axl*.

### *Mir34a*-deficiency promotes migration and M2 polarization in BMDMs

**Fig. 6:**
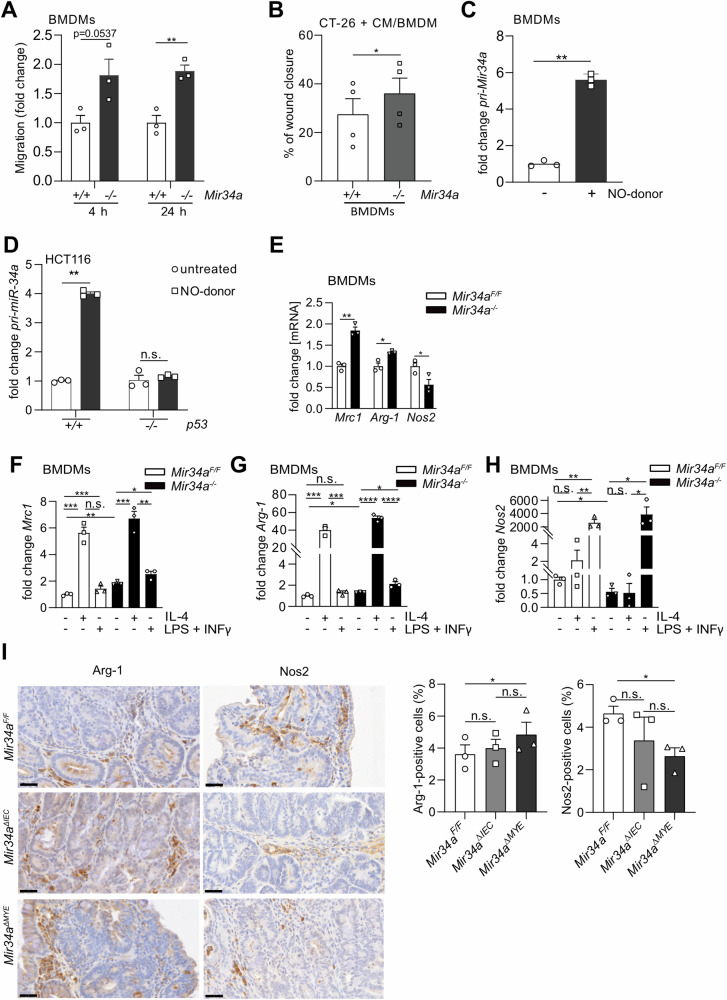
*Mir34a*-deficiency increases migration and polarization towards M2-like macrophages. **A** Fold change of migrated BMDMs after 4 and 24 h (*n* = 3). Assay was performed in triplicate inserts. **B** Percentage of wound closure of CT-26 cells after co-culture with *Mir34a*-proficient or -deficient BMDMs for 24 h (*n* = 4). **C** *pri-Mir34a* expression in *Mir34a*-proficient BMDMs after treatment with 0.5 mM NO-donor (*n* = 3). **D** *pri-miR34a* expression after treatment of HCT116 *p53* wildtype and *p53* knockout cells (*n* = 3) with a 1 mM NO-donor for 24 h. Expression was normalized to *GAPDH*. **E** *Mrc1*; *Arginase-1* and *Nos2* expression in *Mir34a*-proficient and *Mir34a-*deficient BMDMs (*n* = 3). **F** Expression of *Mrc1*; (**G**) *Arginase-1* and (**H**) *Nos2* in *Mir34a*-proficient and *Mir34a-*deficient BMDMs after treatment with IL-4 or LPS + INFγ for 4 h (*n* = 3). **I** Immunohistochemical analysis of Arginase-1 (Arg-1) and Nos2 in CACs of *Mir34a*^*F/F*^, *Mir34a*^*ΔIEC*^ and *Mir34a*^*ΔMYE*^ mice (*n* = 3 mice per genotype). Scale bar represent 40 µm. (**A**–**I**) Students *t*-test was used to determine significance. Values represent the mean ± SEM with *p* < 0.05*; *p* < 0.01**; *p* < 0.001*** and *p* < 0.0001****.

On the functional level, deletion of *Mir34a* in BMDMs resulted in increased migration when compared to *Mir34a*-proficient BMDMs (Figs. 6A and S7A). When murine CT-26 CRC cells were co-cultured with *Mir34a*-deficient BMDMs their migration was increased when compared to co-culture with *Mir34a*-proficient BMDMs (Figs. 6B and S7B).

Next, we hypothesized that *Mir34a* expression might be induced by NO, which is released by different immune cells during inflammation [62], as it has been reported that p53 is activated by NO in a CAC model [38]. Indeed, treatment of BMDMs with the NO–donor spermine nonoate induced the expression of *pri-Mir34a* (Fig. 6C). Furthermore, the induction of *pri-miR-34a* by NO is p53-dependent, since *p53*-proficient HCT116 cells, but not *p53*-deficient HCT116 CRC cells showed an induction of *pri-miR-34a* after addition of an NO-donor (Fig. 6D). These results provide a model as to how myeloid *Mir34a* expression is induced under physiological conditions.

Next, we aimed to confirm the increase in M2-like macrophages in *Mir34a*-deficient CACs detected by scRNA-Seq (see Fig. 5G). In *Mir34a*-deficient BMDMs a significant elevation of *Arginase-1* and *Mrc1* and a significant decrease of *Nos2* mRNA levels was detected when compared to *Mir34a*-proficient BMDMs (Fig. 6E). Treatment of BMDMs with IL-4 led to significantly elevated levels of *Arg-1* and *Mrc1* in BMDMs compared to their untreated control (Fig. 6F–G). BMDMs treated with LPS + INFγ showed increased *Nos2* expression compared to untreated control BMDMs (Fig. 6H). Unexpectedly, after IL4-treatment, the induction of *Mrc1* was significantly less pronounced in *Mir34a*-deficient BMDMs when compared to *Mir34a*-proficient BMDMs, whereas the repression of *Nos2* was more pronounced in *Mir34a*-deficient BMDMs (Fig. S7C). After LPS/IFNγ treatment *Nos2* was more induced in *Mir34*-deficient BMDMs, whereas the minor induction of *Mrc1* and *Arg1* was not affected by *Mir34a* deletion (Fig. S7D). In addition, using immunohistochemical detection of protein markers, we observed, that the number of stromal cells displaying expression of the M2-marker *Arginase-1* was significantly elevated in *Mir34a*^*ΔMye*^ CACs, whereas stromal cells positive for the M1 marker *Nos2* were decreased when compared to control CACs (Fig. 6I). In the iKAP mouse model the expression of M2 markers *Arg1* and *Mrc1* increased from normal colon (NC) towards non- and low-invasive tumors (TA and T1) and reached its highest level in tumors that invade through the serosa and the outer intestinal wall (T4) and liver metastases (Fig. S8A, S8B), whereas the M1 marker *Nos2* showed the opposite expression pattern (Fig. S8C). In human CRC patients, high expression of M2 makers *ARG1* and *MRC1* was significantly associated with poor survival, whereas high expression of the M1 marker *NOS2* was significantly associated with good survival in the majority of ten analyzed CRC patient cohorts (Fig. S8D–F). Overall, these results imply that *Mir34a* may prevent CAC migration and invasion by inhibiting polarization of macrophages towards M2.

### Reduction of tumor burden in *Mir34a*^*ΔMye*^ CACs by concomitant deletion of *Csf1r*

**Fig. 7:**
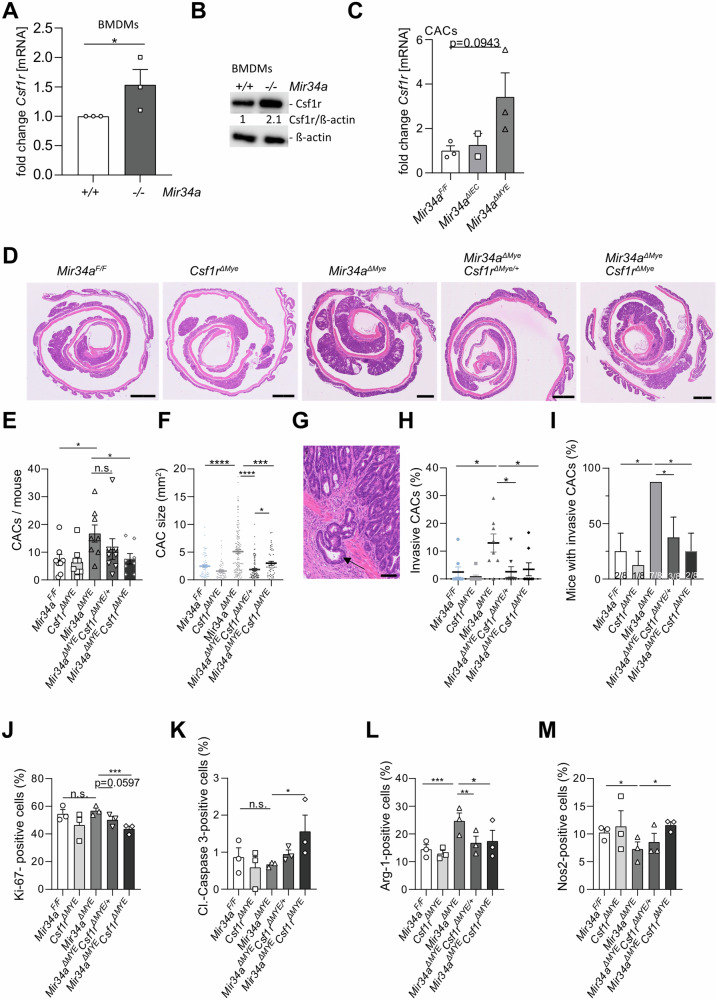
Concomitant deletion of *Csf1r* reverses the effects of myeloid-specific *Mir34a*-deletion on CACs. **A** *Csf1r* expression in *Mir34a*-proficient and *Mir34a*-deficient bone-marrow derived macrophages (BMDMs) (*n* = 3 mice per genotype). **B** Western blot analysis of Csf1r in *Mir34a*-proficient and *Mir34a*-deficient BMDMs. **C** *Csf1r* expression in CACs of *Mir34a*^*F/F*^, *Mir34a*^*ΔIEC*^ and *Mir34a*^*ΔMYE*^ mice (*n* = 3 mice per genotype). **D** Histology of CACs with indicated genotypes, scale bars are 1600 µm. **E** CAC incidence in *Mir34a*^*F/F*^, *Csf1r*^*ΔMye*^, *Mir34a*^*ΔMye*^, *Mir34a*^*ΔMye*^*Csf1r*^*ΔMye/+*^ and *Mir34a*^*ΔMye*^*Csf1r*^*ΔMye*^ mice (*n* = 8 mice per group). **F** CAC size of mice with indicated genotypes, CAC areas measured by computerized image analysis (*n* = 8 mice per group). **G** Example of CAC invasion in *Mir34a*^*ΔMye*^ mice. Scale bars are 100 µm. **H** Percentage of invasive CACs of indicated genotypes. **I** Number of mice with invasive CACs/total number of mice of indicated genotype. **J** Immunohistochemical quantification of Ki-67, (**K**) Cleaved-Caspase 3, (**L**) Arginase-1 (Arg-1) and (**M**) Nos2 in CACs of indicated genotypes (*n* = 3 per genotype). (**A**, **B**, **C**, **E**, **F**, **H**, **I**, **J**, **K**, **L**, **M**) Students *t*-test was used to determine significance. Values represent the mean ± SEM with *p* < 0.05*; *p* < 0.01**; *p* < 0.001*** and *p* < 0.0001****.

The receptor tyrosine kinase Csf1r plays an important role in macrophage recruitment and has been validated as a miR-34a target in CRC cells relevant for the formation of murine intestinal adenomas and human CRCs [18, 19]. As shown above, our scRNA-Seq analysis detected increased expression of *Csf1r* and its ligand *CSF1* in *Mir34a*^*ΔMye*^ vs. *Mir34a*^*F/F*^ macrophages and neutrophils (Figs. 4K and 5J). In *Mir34a*-deficient BMDMs, we could observe an increased expression of *Csf1r* on mRNA and protein levels when compared to *Mir34a*-proficient BMDMs (Fig. 7A, B; uncropped Western blot membrane: see Supplementary Material). The number of cells positive for the macrophage marker F4/80 was significantly increased (Fig. S9A) in CACs of *Mir34a*^*ΔMye*^ mice when compared to *Mir34a*^*ΔIEC*^ and *Mir34a*^*F/F*^ mice. Csf1r positive, stromal cells were also more abundant in the CAC stroma of *Mir34a*^*ΔMye*^ mice when compared to *Mir34a*^*ΔIEC*^ and *Mir34a*^*F/F*^ mice (Fig. S9B), suggesting that the *Mrc1*^*+*^ macrophage subtype identified by scRNA-Seq might contribute to this cell population (Fig. 5K). Furthermore, an increase of *Csf1r* mRNA expression in CACs of *Mir34a*^*ΔMye*^ mice was observed (Fig. 7C). In addition, CSF1R expression was highest in the CMS4 and CRISB molecular subtypes of CRCs in cohorts of CRC patients (Fig. S9C). These subtypes are associated with EMT, metastasis and poor prognosis [36, 63]. Furthermore, scRNA-Seq analysis of human CRCs [54] (GSE81861) revealed that *CSF1R* is predominantly expressed in myeloid cells (Fig. S9D). In the iKAP mouse model the expression of *Csf1r* was lowest in normal colon (NC), increased in non- and low-invasive tumors (TA and T1), and reached its highest level in tumors that invade through the serosa and the outer intestinal wall (T4) and was still elevated in the corresponding liver metastases (Fig. S9E). In addition, we analyzed the expression of *Csf1r* in publicly available RNA-Seq data from the KPN model of metastatic CRC [64], which combines mutant K-*Ras* (K), *p53* (P) deletion and *NOTCH1* (N) activation. KP and KPN mice develop lymph node and liver metastases, whereas AP and APN mice do not. The expression of *Csf1r* was elevated in KP and KPN mice when compared to AP and APN mice (Fig. S9F). Taken together, these results imply that the elevated expression of *Csf1r* caused by *Mir34a*-deficiency may play a major role in tumor progression via TME—tumor cell interactions in the mouse model analyzed here.

In order to determine whether the observed upregulation of *Csf1r* in macrophages is required for the effects of *Mir34a-*deficiency we generated *Mir34a*-deficient mice with concomitant heterozygous and homozygous deletion of *Csf1r* in myeloid cells. *Csf1r*^*ΔMye*^ mice were used as additional controls. Interestingly, concomitant *Csf1r* deletion—heterozygous as well as homozygous—reduced the number of CACs when compared to *Mir34a*^*ΔMye*^ mice (Fig. 7D, E). *Csf1r* deletion alone had no effect when compared to *Mir34a*^*fl/fl*^ mice. Furthermore, the increased size of CACs with a myeloid *Mir34a* deletion was reduced by concomitant *Csf1r* inactivation (Fig. 7F). In *Mir34a*^*ΔMye*^ mice we observed in almost every mouse at least one invasive CAC. Therefore, the percentage of invasive CACs as well as mice with invasive CACs was significantly increased in *Mir34a*^*ΔMye*^ mice when compared to *Mir34a*^*fl/fl*^ or *Csf1r*^*ΔMye*^ mice, and was significantly decreased by concomitant deletion of *Csf1r* when compared to *Mir34a*^*ΔMye*^ mice (Figs. 7G–I and S10A).

Next, we determined whether inactivation of *Csf1r* in this context also influences proliferation and apoptosis. Concomitant homozygous deletion of *Csf1r* and *Mir34a* indeed reduced the number of Ki-67-positive cells significantly, whereas the number of Cleaved-Caspase 3 positive cells increased (Figs. 7J, K and S10B) indicating that deletion of *Csf1r* in *Mir34a*-deficient myeloid cells reduces proliferation and increases apoptosis. Concomitant myeloid-specific deletion of *Csf1r* with *Mir34a* resulted in a reduction of cells expressing the M2 marker Arginase-1 in CACs when compared with CACs in *Mir34a*^*ΔMye*^ mice. *Nos2,* a M1 marker, showed the opposite pattern of expression. Concomitant homozygous deletion of *Csf1r* and *Mir34a* increased the number of Nos2-positive cells significantly to the level present in CACs of *Mir34a*^*fl/fl*^ and *Csf1r*^*ΔMye*^ mice (Figs. 7L, M and S10B). Taken together, these results demonstrate that the increase in *Csf1r* expression is a required mediator of the effects of *Mir34a* inactivation on myeloid cells. The results imply that Mir34a inhibits CAC progression and M2-like polarization, at least in part, by decreasing *Csf1r* expression levels.

### Inhibition of Csf1r in *Mir34a*^*ΔMye*^ mice reduces tumor burden

**Fig. 8:**
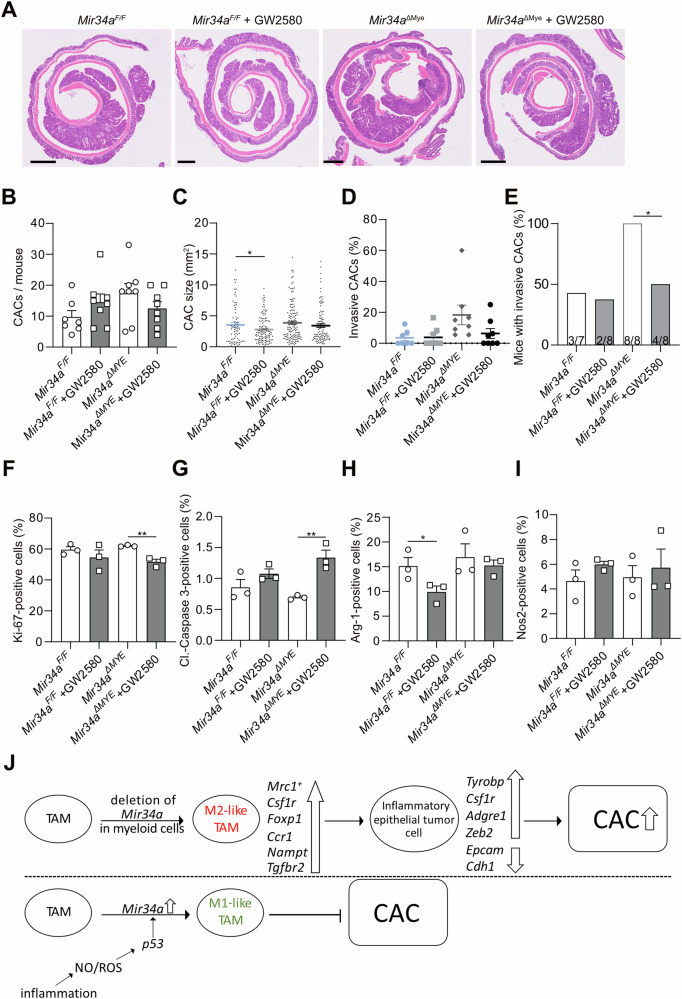
Effects of chemical inhibition of *Csf1r* in mice with myeloid-specific deletion of *Mir34a* deletion. **A** Histology of CACs with indicated genotypes, scale bars are 1600 µm. **B** CAC incidence in *Mir34a*^*F/F*^, *Mir34a*^*F/F*^ + GW2580, *Mir34a*^*ΔMye*^ and *Mir34a*^*ΔMye*^ + GW2580 mice (*n* = 7–8 mice per group). **C** CAC size of mice with indicated genotypes, CAC areas measured by computerized image analysis (*n* = 7-8 mice per group). **D** Percentage of invasive CACs of indicated genotypes. **E** Number of mice with invasive CACs/total number of mice of indicated genotype. **F** Immunohistochemical quantification of Ki-67, (**G**) Cleaved-Caspase 3, **H** Arginase-1 (Arg-1) and (**I**) Nos2 in CACs of indicated genotypes (*n* = 3 per genotype). **J** Model summarizing the results and main conclusions. (**B**, **C**, **E**–**I**) Students *t*-test was used to determine significance. Values represent the mean ± SEM with *p* < 0.05* and *p* < 0.01**.

Since CAC-associated macrophages with *Mir34a*-deficiency displayed an elevated level of *Csf1r* expression (Fig. 5J, K), they may be dependent on Csf1r signaling and therefore especially sensitive to its inhibition. Therefore, we asked whether therapeutic inhibition of Csf1r has a preferential effect on *Mir34a*-deficient CACs. To induce CACs, mice were subjected to AOM/DSS as described above and received the small molecule Csf1r inhibitor GW2580 from day 60 onwards. In *Mir34a*-deficient mice treatment with GW2580 significantly decreased the number of CACs and invasiveness (Figs. 8A, B, D and E; S11A). In wild-type mice treatment with GW2580 significantly reduced CAC size although the number of CACs showed a minor increase (Fig. 8B, C). GW2580 had no effect on invasion of CACs in wild-type mice (Fig. 8D, E). Treatment of *Mir34a*^*ΔMye*^ mice with the GW2580 resulted in decreased proliferation and increased apoptosis in CACs, whereas it had no effect in *Mir34a*^*fl/fl*^ mice (Fig. 8F and G; S11B). GW2580 significantly decreased the number of Arg-1-positive cells in CACs in wild-type mice, but in CACs of *Mir34a*^*ΔMye*^ mice no significant effect was evident (Fig. 8H). For both genotypes the number of Nos2-positive cells increased with GW2580 treatment by ~2%. However, this effect did not reach significance (Figs. 8I and S11B). Taken together, these results demonstrate that CACs with *Mir34a*-deficient myeloid cells and therefore increased *Csf1r* expression levels are sensitive to treatment with a Csf1r inhibitor. In addition, these results partially agree with the observations obtained by genetic inactivation of *Csf1r* in *Mir34a-*deficient myeloid cells and further confirm Csf1r-inhibition as an important mediator of non-tumor-cell autonomous suppression of CACs by miR-34a.

## Discussion

Here we show that deletion of *Mir34a* in myeloid cells promotes CAC formation and progression (see summarizing model in Fig. 8J). Therefore, *Mir34a* functions as a non-cell autonomous suppressor of CAC formation in tumor-associated myeloid cells. Notably, myeloid-specific inactivation of *Mir34a* increased CAC invasiveness to a larger extent than intestinal epithelial cell-specific deletion of *Mir34a*. Among other *Mir34a* target mRNAs, *Csf1r* was upregulated in *Mir34a*-deficient myeloid cells in CACs. In mice with inactivation of *Mir34a* in myeloid cells concomitant *Csf1r* deletion as well as therapeutic inhibition of Csf1r resulted in decreased proliferation, elevated apoptosis, less invasiveness of CACs and decreased macrophage polarization towards M2. Therefore, *Csf1r* is a required mediator of the effects of *Mir34a* inactivation in tumor-associated myeloid cells.

We found that deletion of *Mir34a* in myeloid cells increased the number of macrophages and neutrophils. In particular, the number of *Mrc1*^*+*^ and *CDK8*^*+*^ macrophages was highly increased in *Mir34a*^*ΔMye*^ CACs. Since these subtypes of macrophages are known to promote cancer [65, 66], their increased abundance may also promote progression of CACs. In a study of lung cancer, it was shown that under hypoxic stress tumor cells suppress exosomal miR-101 [65]. This suppression of miR-101 enhanced CDK8 in macrophages which promoted the expression of pro-inflammatory cytokines IL1A and IL6 in macrophages. Impairment of those cytokines suppressed tumor cell growth in vitro and in vivo. Interestingly, in a study by Zhang and colleagues, two TAM subtypes expressing *Maf* and *Mgl2* were identified which are sensitive to anti-Csf1r treatment [54]. These genes were predominantly expressed in the *Mrc1*^*+*^ subtype of TAMs, which also expressed *Csf1r* and therefore might also be especially sensitive to Csf1r treatment. Additionally, Zhang et al. compared the scRNA-Seq data from murine and human CRCs and concluded that they are similar [54] suggesting that the new therapeutics or druggable pathways identified here in murine CRCs may be transferred to human.

We showed that *Csf1r* deletion or inhibition reprogrammed the macrophages from M2-like towards M1-like, especially in *Mir34a*^*ΔMye*^ mice. Lv et al. showed that the treatment of human M2-like BMDMs with the CSF1R inhibitor PBX17 also reprograms them to M1-like macrophages [67]. They co-cultured human CRC organoids with M2- macrophages and CD8^+^ T-cells and showed that CSF1R inhibition reduces the viability of CRC organoids. However, CSF1R inhibition had no effect on CRC organoid viability without co-culture. These findings indicate that CSF1R inhibition promotes an anti-tumor immune response of CD8^+^ T cells by facilitating the reprogramming of M2-like macrophages towards M1-like macrophages. Consistently, CSF1R inhibition enhanced the therapeutic activity of a PD-1-specific mAb in subcutaneous xenograft mouse models of CRC by improving the infiltration and activation of CD8^+^ T cells in tumors [67]. Since PD-L1 is a miR-34a target [68, 69], which is expressed in macrophages, multimodal therapy studies with Csf1r and PD-1 inhibitors in *Mir34a*^*ΔMye*^ CACs should be performed in the future.

It has been reported that *p53*-deficient mice display an increased risk of developing severe chronic inflammation ([70]; reviewed in ref. [71]) and CACs when exposed to DSS ([72], reviewed in ref. [73]) and that p53 inhibits M2-like polarization [74]. In the *APCmin* mouse model of FAP/familial adenomatous polyposis deletion of *p53* in myeloid cells leads to a higher level of inflammatory cytokines, increased M2-like polarization of TAMs and tumor progression, whereas p53 activation had the opposite effects [37]. Therefore, p53 functions as a non-cell autonomous tumor suppressor in macrophages (also reviewed in refs. [38, 73]). Here, inactivation of the p53 target gene *miR-34a* in myeloid cells associated with CACs had similar effects. Therefore, our results imply that *Mir34a* presumably represents an important mediator of the effects of p53 on macrophages and of the suppression of CAC formation by p53.

During inflammation NO is released from macrophages. Notably, NO was shown to activate p53 [38]. Therefore, *Mir34a* is presumably activated by p53 during inflammation and may direct macrophage polarization towards an M1-like state. In contrast, loss of *p53* or, as shown here, *Mir34a* inactivation in macrophages enhances their polarization towards an M2-like state. We recently showed that *miR-34a* expression is induced by ROS donors, such as curcumin and H_2_0_2_, via the KEAP/NRF2 pathway in a p53-independent manner [75]. Since inflammation is known to generate ROS (reviewed in ref. [76]), it is conceivable that miR-34a is induced by ROS under such circumstances. Therefore, miR-34a may also function to suppress M2-like-polarization in the absence of p53 activation.

Here, the expression of *Csf1r*, which is a conserved miR-34a target mRNA [18, 19], was elevated in macrophages, neutrophils and dendritic cells in *Mir34a*^*ΔMye*^ CACs. Consistently, the presence of CSF1R-positive macrophages is associated with poor survival [11]. We could show that Csf1r inhibition or deletion in myeloid cells decreases the growth and progression of CACs. Therefore, Csf1r-positive macrophages presumably represent important therapeutic targets. Furthermore, CACs harboring *Mir34a*-deficient myeloid cells with increased *Csf1r* expression were more sensitive to treatment with the Csf1r-inhibitor GW2580 than CACs of wild-type mice. This presumably resulted from an increased dependency on Csf1r signaling. Inhibitors of CSF1R or its ligand CSF1 interfere with tumor-promoting TAMs or other myeloid cells in the TME and are currently being tested in clinical trials for treatment of several types of malignancies, such as CRC, melanoma or ovarian cancer (reviewed in refs. [11, 77]). Here we did not study the effect of myeloid *Csf1r* deletion or Csf1r inhibition on overall survival of mice, because we focused on the differences in CAC characteristics at a specific time point. The effect on overall survival should be determined in the future.

Notably, our scRNA-Seq analysis revealed a CAC cell subtype, designated as inflammatory cancer cells, which was almost exclusively present in *Mir34a*^*ΔMye*^ CACs. These cells expressed pro-inflammatory genes, such as Cd74, which contributes to inflammation-driven epithelial cell regeneration in IBD [78]. Furthermore, CD74 was shown to promote a pro-inflammatory tumor microenvironment in pancreatic cancer by inducing the secretion of S100A8/A9 via activation of TRAF6-NF-κB [44]. *S100a8* and *S100a9* were also highly expressed in the inflammatory cluster of *Mir34a*-deficient CACs. These proteins contribute to the progression of inflammatory diseases and cancer [45]. Interestingly, the inhibition of S100A8/A9 with chemical inhibitors decreased tumor burden in the AOM/DSS-induced mouse model of CAC [79]. Therefore, increased levels of Cd74 and S100a8/9 in the inflammatory CAC cell subtype may drive migration and invasion in myeloid *Mir34a*-deficient CACs.

Furthermore, the inflammatory cancer cell subtype identified here expressed high levels of the EMT transcription factor Zeb2, particularly in *Mir34a*^*ΔMye*^ CACs. Interestingly, it has been shown that mice with intestinal epithelial cell-specific, ectopic expression of *Zeb2* develop invasive CRCs, which are driven by myeloid cell-induced inflammation [80]. Thus, the increased expression of Zeb2 may also play an important role in the enhanced invasion of *Mir34a*^*ΔMye*^ CACs.

Among the M2-like macrophage-derived factors with a known role in tumor progression, *Pdgfc* and *Il10* were upregulated in *Mir34a*-deficient macrophages, particularly in the Mrc1^+^ subtype. Pdgfc promotes angiogenesis (reviewed in ref. [59]) and Il10 contributes to tumor immune evasion (reviewed in ref. [59]), suggesting that these factors might contribute to the enhanced progression of *Mir34a*^*ΔMye*^ CACs. Our results also demonstrate that the semaphorin signaling is elevated in *Mir34a*^*ΔMye*^ CACs. The Sema4d ligand, which represents a potential miR-34a target was upregulated in *Mir34a*-deficient macrophages and neutrophils. It has been shown that TAM-derived Sema4d promotes angiogenesis, invasion, and metastasis in breast and gastric cancer [81, 82]. Furthermore, elevated expression of Sema4D in macrophages was associated with poor prognosis in ovarian cancer and facilitated the differentiation of macrophages toward the M2-like lineage [83]. Therefore, the elevated semaphorin signaling might also contribute to the enhanced progression of *Mir34a*^*ΔMye*^ CACs. However, to achieve a better understanding of the crosstalk between myeloid *Mir34a*-deficient stromal cells and CAC cells and to identify myeloid cell-secreted factors that promote tumor progression, further investigations are needed.

Matrix metalloproteinase 9 (Mmp9) showed elevated expression levels in *Mir34a*^*ΔMye*^ neutrophils and represents a known miR-34 target [84]. Neutrophils release Mmp9, which degrades collagen and thereby remodels the ECM to promote tumor cell invasion and metastasis [49]. Moreover, Mmp9 promotes VEGF activation and tumor angiogenesis [85]. Interestingly, neutrophils are the only cells that can release Mmp9 from its endogenous inhibitors, the tissue inhibitors of metallo-proteinases [86]. Thus, Mmp9 in neutrophils may represent a potential therapeutic target in cancer.

Taken together, our study provides evidence that myeloid *Mir34a* prevents CAC progression by inhibiting M2-like polarization of TAM which is, at least in part, mediated by the downregulation of Csf1r signaling. Furthermore, we identified multiple, additional hallmarks of CACs harboring *Mir34a*-deficient myeloid cells, such as increased expression of *Mmp9* in neutrophils and elevated expression of *Zeb2* in an inflammatory subtype of CAC tumor cells. In *Mir34a*-deficient macrophages the miR-34a target mRNA encoding the Axl receptor-tyrosine-kinase, an already approved drug target [87], was upregulated. Also, *engulfment and cell motility protein 1* (*ELMO1)* displayed increased expression in *Mir34a*-deficient macrophages. As shown previously, elevated *ELMO1* expression in stromal cells may promote CRC progression and is associated with poor survival [88]. Furthermore, the predicted *Mir34a* target *protein kinase C beta type* (*Prkcb)* was elevated in macrophages and neutrophils of *Mir34a*^*ΔMye*^ CACs. Interestingly, *Prkcb* was reported to have tumor-promoting properties in breast cancer [89]. This may also apply to CACs. Targeting these and additional, deregulated factors in myeloid cells within the TME identified in this study may improve therapy of CAC in the future.

## Supplementary information

Supplemental Methods, Material, Figures, Tables

Extended data 1

## Data Availability

Single cell RNA-Seq data from this study has been deposited at NCBI GEO GSE253907. Codes were implemented in R (version 4.1.2) and python (version 3.10.0) and are deposited in https://github.com/MatjazRokavec/miR34a_MYE_KO_scRNA-seq. The RNA-Seq data from the iKAP mouse model [46] is available at https://trace.ncbi.nlm.nih.gov/Traces/?view=study&acc=SRP097890. The RNA-Seq data from the KPN mouse model [64] is available at https://www.ebi.ac.uk/ena/browser/view/PRJEB38364.
