# Supplementary material for: Myeloid Mir34a suppresses colitis-associated colon cancer: characterization of mediators by single-cell RNA sequencing
Source: Cell Death Differ. 2024 Oct 18;32(2):225–41. doi: 10.1038/s41418-024-01380-9 (PMC11802797; doi:10.1038/s41418-024-01380-9)
Supplement: Supplementary file 1 — Supplemental Methods, Material, Figures, Tables [file 41418_2024_1380_MOESM1_ESM.pdf]

List of contents for Supplementary Material:

Supplementary Methods

Method S1. Quantitative real-time PCR

Method S2. Primary cell culture (BMDMs)

Method S3. Cell culture

Method S4. Western blot analysis

Method S5. Histological analysis and tumor size/number evaluation

Method S6. Immunohistochemical (IHC) staining

Method S7. Modified Boyden chamber assay

Method S8. Scratch assay

Supplementary Tables

Table S1: Kits

Table S2: Antibodies

Table S3: Oligonucleotides

Table S4: MASC-based statistics for the differences of proportions of cell types between Mir34a$^{\Delta Mye}$ and Mir34a$^{F/F}$ mice

Supplementary Figures

Supplementary Figure S1. Related to Figure 2. scRNA-Seq analysis of CACs and their microenvironment in mice with myeloid-specific deletion of *Mir34a*.

Supplementary Figure S2. Related to Figure 3. scRNA-Seq analysis of CAC cells in mice with myeloid-specific deletion of *Mir34a*.

Supplementary Figure S3. Related to Figure 3. scRNA-Seq analysis of CAC cells in mice with myeloid-specific deletion of *Mir34a*.

Supplementary Figure S4. Related to Figure 4. scRNA-Seq analysis of CAC-associated neutrophils in mice with myeloid-specific deletion of *Mir34a*.

Supplementary Figure S5. Related to Figure 5. scRNA-Seq analysis of CAC-associated macrophages and monocytes in mice with myeloid-specific deletion of *Mir34a*.

Supplementary Figure S6. Related to Figure 5. scRNA-Seq analysis of CAC-associated macrophages and monocytes in mice with myeloid-specific deletion of *Mir34a*.

Supplementary Figure S7. Related to Figure 6. *Mir34a*-deficiency increases migration and polarization towards M2-like macrophages.

Supplementary Figure S8. Related to Figure 6. *Mir34a*-deficiency increases migration and polarization towards M2-like macrophages.

Supplementary Figure S9. Related to Figure 7. Concomitant deletion of *Csf1r* reverses the effects of myeloid-specific *Mir34a*-deletion on CACs.

Supplementary Figure S10. Related to Figure 7. Concomitant deletion of *Csf1r* reverses the effects of myeloid-specific *Mir34a*-deletion on CACs.

Supplementary Figure S11. Related to Figure 8. Effects of chemical ínhibition of *Csf1r* in mice with myeloid-specific deletion of *Mir34a* deletion.

Original Western blots (uncropped membranes)

Supplementary Methods

Method S1. Quantitative real-time PCR

RNA was isolated using RNeasy Mini Kit (Qiagen) and cDNA was prepared using Verso cDNA Kit (Thermoscientific) following manufacturer's instructions. A mix containing Fast SYBR Green Mastermix (Applied Biosystems), dH2O, cDNA and the indicated primers (listed in Table S2) was prepared and samples were run on a Light Cycler 480 (Roche Diagnostics) using the program IDEAS 2.0. Samples were evaluated using the ΔΔCt method (1) and normalized to *cyclophilin* expression.

Method S2. Primary cell culture (BMDMs)

Femur and tibia of mice were collected and bone-marrow was flushed with HBSS containing 10% FBS. The flush-through was filtered, centrifuged and cells were seeded in 10 ml RPMI medium containing 20% L929 conditioned medium. 24 hours later supernatant was collected, centrifuged and re-seeded followed by a change of medium to RPMI medium supplemented with 10% L929 conditioned medium on day 5 after isolation. 48 hours later cells were seeded. One day after seeding BMDMs were treated with 10 ng/ml murine IL-4 (Peprotech; #214-14), 100 ng/ml LPS (Sigma-Aldrich; # L4391) and 5 ng/ml murine INFγ (Peprotech; #315-05) for 4 hours or with 0.5 nM NO donor (Spermine Nonoate; Cyman Chemicals; #Cay82150-10) for 24 hours.

Method S3. Cell culture

The murine colorectal cancer cell-line CT-26 was cultured in RPMI1640 medium and the human colorectal cancer cell-line HCT116 was cultured in Dulbecco's modified Eagle medium (DMEM). HCT116 cells were treated with 1 mM NO donor (Spermine Nonoate) for 24 hours.

Method S4. Western blot analysis

Isolated CACs were lysed using RIPA buffer containing protease and phosphatase inhibitor cocktail tablets (Roche). Protein concentration was measured using the BCA Protein Assay Kit (Thermo Scientific) according to manufacturer's instructions. Western blot analysis was performed according to a standard protocol. In brief, proteins were separated on 12% sodium dodecyl sulfate–acrylamide gels and transferred to Immobilon-P membranes. Membranes were incubated with first antibodies and, after washing, then with horseradish-peroxidase coupled secondary antibodies. Antibodies are listed in Table S1. Signals were generated with enhanced chemiluminescent substrate (WBKLS0100; Merck Millipore) and visualized using an

Odyssey Fc imaging system (LI-COR). Data were evaluated using Image Studio Ver. 5.2.

Method S5. Histological analysis and tumor size/number evaluation

The colon was opened longitudinally and rolled to form a “swiss roll” as described previously (2). Tissues were fixed overnight in 4% paraformaldehyde/PBS (Santa Cruz), dehydrated, embedded in paraffin and 2 µm serial sections were obtained. Number, size and invasiveness of CACs were evaluated on hematoxylin and eosin (H&E) stained sections by using AxioVision Rel. 4.8 (Zeiss) or ImageJ software.

Method S6. Immunohistochemical (IHC) staining

Tissue sections were deparaffinized, rehydrated and boiled in antigen retrieval solution (Dako). After blocking, primary antibodies were applied and incubated at 4°C over-night or 1 hour at room temperature (RT). Using DAB (3,3´-Diaminobenzidine) (Dako) the bound antibodies were visualized and counterstained with hematoxylin (Vector). Sections were scanned with a Vectra® Polaris™ Automated Quantitative Pathology imaging system and quantified using Image J software. Antibodies are listed in Table S2.

Method S7. Modified Boyden chamber assay

50,000 BMDMs per Transwell-insert were seeded in culture medium for one day into 24-well Transwell chambers (Greiner Bio One) with an 8 µM pore size. 2 hours prior to the assay the medium was changed to low FBS (0.5 %). The lower chamber was filled with normal growth medium and the Transwell-Inserts (upper chamber) with BMDMs were placed on top. After 4 and 24 hours the Transwell-inserts were removed, BMDMs were fixed with 4% PFA, stained with 0.1% crystal violet and non-migratory cells on the top filter were removed. The cell number was quantified using Image J software.

Method S8. Scratch assay

70 µl CT-26 cell suspension ($1x10^6$ cells/ml) per Insert side (Ibidi; #80209) and 200,000 BMDMs were seeded per Transwell. 2 hours before co-cultivation 10 ng/mg Mitomycin C (Sigma-Aldrich; #M0503) was added to CT-26 cells. Followed by co-cultivation of CT-26 cells with *Mir34a*-proficient or *Mir34a*-deficient BMDMs for 24 hours. The percentage of wound closure was measured with AxioVision SE (Zeiss).

Supplementary Tables

Table S1. Kits

Name	Catalog Number	Company	Use
Impress®HRP Horse Anti-Rabbit IgG Polymer Kit	MP-7401	Vector laboratories Inc.	IHC
M.O.M. ® (Mouse on Mouse) Immunodetection Kit, Basic	BMK-2202	Vector laboratories Inc.	IHC
VECTASTAIN®Elite® ABC Universal Kit, Peroxidase, R.T.U (Horse Anti-Mouse/Rabbit IgG)	PK-7200	Vector laboratories Inc.	IHC
Verso cDNA Synthesis Kit	AB-1453/B	Thermo Fisher Scientific	PCR
Tumor Dissociation kit, mouse	# 130-096-730	Miltenyi Biotec	Sc-RNAseq
MACS Dead Cell Removal Kit	# 130-090-101	Miltenyi Biotec	Sc-RNAseq

IHC: Immunohistochemistry

Table S2. Antibodies

Name	Species	Catalog Number	Company	Use	Dilution	Source
F4/80	mouse	ab6640	Abcam	IHC	1:100	rat
Csf1r	mouse/human	sc-692	Santa Cruz Biotechnology	WB	1:500	rabbit
Csf1r	mouse	SAB4500500	Sigma Aldrich	IHC	1:1200	rabbit
Arginase-1	mouse/rat	sc-271430	Santa Cruz Biotechnology	IHC	1:200	mouse
Nos2 (iNOS)	mouse/rat /human	sc-7271	Santa Cruz Biotechnology	IHC	1:50	mouse
ß-actin	mouse/human	A2066	Sigma Aldrich	WB	1:1000	rabbit
Cleaved-Caspase 3	mouse	9661S	Cell signaling	IHC	1:200	rabbit
Ki-67	mouse	12202S	Cell signaling	IHC	1:400	rabbit
ECL		WBKLS0100	Millipore	WB		

IHC: Immunohistochemistry; WB: Western blot analysis

Table S3. Oligonucleotides

Name	Sequence (5'-3')	Use
mmu-Cyclophilin R	TTCTGCTGTCTTTGGAACTTTGTC	qPCR
mmu-Cyclophilin F	ATGGTCAACCCCACCGTGT	qPCR
mmu-Arginase-1 F	GTGAAGACGGCAGTGGCT TT	qPCR
mmu-Arginase-1 R	GTCCCTGGCTTATGGTTACCC	qPCR
mmu-Nos2 F	GTTCTCAGCCCAACAATACAAGA	qPCR
mmu-Nos2 R	GTGGACGGGTCGATGTCAC	qPCR
mmu-Csf1r F	TGTCATCGAGCCTAGTGGC	qPCR
mmu-Csf1r R	CGGGAGATTCAGGGTCCAAG	qPCR
mmu-Mrc1 F	TTGGTGGCAATTCACGAGAG	qPCR
mmu-Mrc1 R	GGGAAGGGTCAGTCTGTGTTTG	qPCR
mmu-pri-Mir34a F	CTGTGCCCTCTTGCAAAAGG	qPCR
mmu-pri-Mir34a R	GGACATTCAGGTGAGGGTCTTG	qPCR
mmu-370 (Cre) Fwd	ACCTGAAGATGTTCGCGATTATCT	GT
mmu-370 (Cre) Rev	ACCGTCAGTACGTGAGATATCTT	GT
mmu-144 (Cre) Fwd	CACAGTGCCCACATTATTTAGATA	GT
mmu-321 (Cre) Rev	GTCTTCAACCTCCCAAGCCTT	GT
mmu-Mir34a FWD	ACCTTGCAGGTGCTCAGAAT	GT
mmu-Mir34a R2	TGGAGCTAACGGAGTGTGTG	GT
mmu-Mir34a R4	CTACCCAAGCTCGACGAAGT	GT
mmu-Mir34a R8	TGCAGCACTTCTAGGGCAGT	GT
mmu-Csf1r F	CATGGCTGTGGCCTAGAGA	GT
mmu-Csf1r R	GGACTAGCCACCATGTCTCC	GT
Hsa-pri-miR34a F	CGTCACCTCTTAGGCTTGGA	qPCR
Hsa-pri-miR34a R	CATTGGTGTCGTTGTGCT	qPCR
Hsa-GAPDH F	TGTTGCCATCAATGACCCCTT	qPCR
Hsa-GAPDH R	CTCCACGACGTACTCAGCG	qPCR

qPCR: Quantitative real-time PCR; GT: Genotyping

Table S4. MASC-based statistics for the differences of proportions of cell types between *Mir34a*$^{\Delta Mye}$ and *Mir34a*$^{F/F}$ mice

Cell Type	size	p-value	OR	OR.95.ci.lower	OR.95.ci.upper	adjusted p-value
MAC	4809	6.85344E-21	1.335398572	1.256931348	1.41876412	7.53878E-20
Epithelial	21479	9.17671E-16	0.848625422	0.815326707	0.883284078	5.04719E-15
Erythroid	136	5.19192E-10	0.308434239	0.205658792	0.462570463	1.90371E-09
Neutrophil	2130	0.007776694	1.125982842	1.031817964	1.228741543	0.014257272
T_cells	5737	0.009104276	0.927985194	0.877306032	0.981592009	0.014306719
B_cells	1869	0.1136707	1.077916435	0.982277262	1.182867536	0.156297213
DC	778	0.1657562	1.067035684	0.915202884	1.576764794	0.25582955
Mast	1517	0.2533465	1.080573428	0.906536709	1.308237843	0.3373623
Fibroblast	117	0.4454049	1.151804492	0.801348702	1.65552608	0.544383767
Basophil	88	0.6642342	0.911265769	0.598923866	1.386495643	0.73065762
Endothelial	223	0.9472137	1.008940016	0.775325423	1.312945392	0.9472137

Macrophage cluster	size	p-value	OR	OR.95.ci.lower	OR.95.ci.upper	adjusted p-value
Cdk8 Mac	755	7.78043E-76	5.387452085	4.396489491	6.601775614	5.4463E-75
Spp1 Mac	593	1.10794E-48	0.258276376	0.213101633	0.313027579	3.87779E-48
Monocytes	941	1.66501E-25	0.462954197	0.399926541	0.535914871	3.88503E-25
Nos2 Mac	327	7.46287E-15	0.401763759	0.317076403	0.509070102	1.306E-14
Mrc1 Mac	927	1.40136E-13	1.745593076	1.502599347	2.027882574	1.96191E-13
Nr4a Mac	506	0.001699636	1.350261654	1.117773538	1.631105575	0.001982909
Mt Mac	549	0.04849986	1.198673979	1.000561315	1.43601326	0.04849986

Neutrophil cluster	size	p-value	OR	OR.95.ci.lower	OR.95.ci.upper	adjusted p-value
TAN5	84	2.44E-23	43.39533804	10.64416122	176.9190364	2.19667E-22
TAN1	326	5.74E-07	0.544459907	0.427593986	0.693266508	2.58484E-06
TAN2	207	1.05E-04	0.563011682	0.419373152	0.755847515	0.000314771
Neutro3	411	2.39E-02	1.281256361	1.032872948	1.589370574	0.053688353
Nautro1	261	4.83E-01	1.096882052	0.846809308	1.420804203	0.87019596
TAN6	29	6.09E-01	1.211626428	0.580016387	2.531029555	0.91296315
Neutro2	551	6.23E-01	1.049578245	0.865384283	1.272977244	0.801075471
TAN3	204	6.67E-01	0.938667706	0.703636304	1.252205238	0.750192075
TAN4	125	8.44E-01	0.964388043	0.672137114	1.38371216	0.8439393

Epithelial cluster	size	p-value	OR	OR.95.ci.lower	OR.95.ci.upper	adjusted p-value
Inflammatory	1869	0	16.09551983	13.58546507	19.0693363	0
Lgr5	4598	4.42806E-67	0.543536564	0.506846624	0.582882438	1.32842E-66
Ribosomal	755	9.50519E-35	2.57663087	2.202330431	3.014546089	1.90104E-34
Krt20	5267	7.04397E-26	0.702603649	0.657718455	0.750551978	1.0566E-25
Ki67	1271	4.10902E-10	0.690649937	0.614281283	0.776512889	4.93083E-10
Peak1	2911	0.0004768	0.866625834	0.799657069	0.939203022	0.0004768

Supplementary Figures

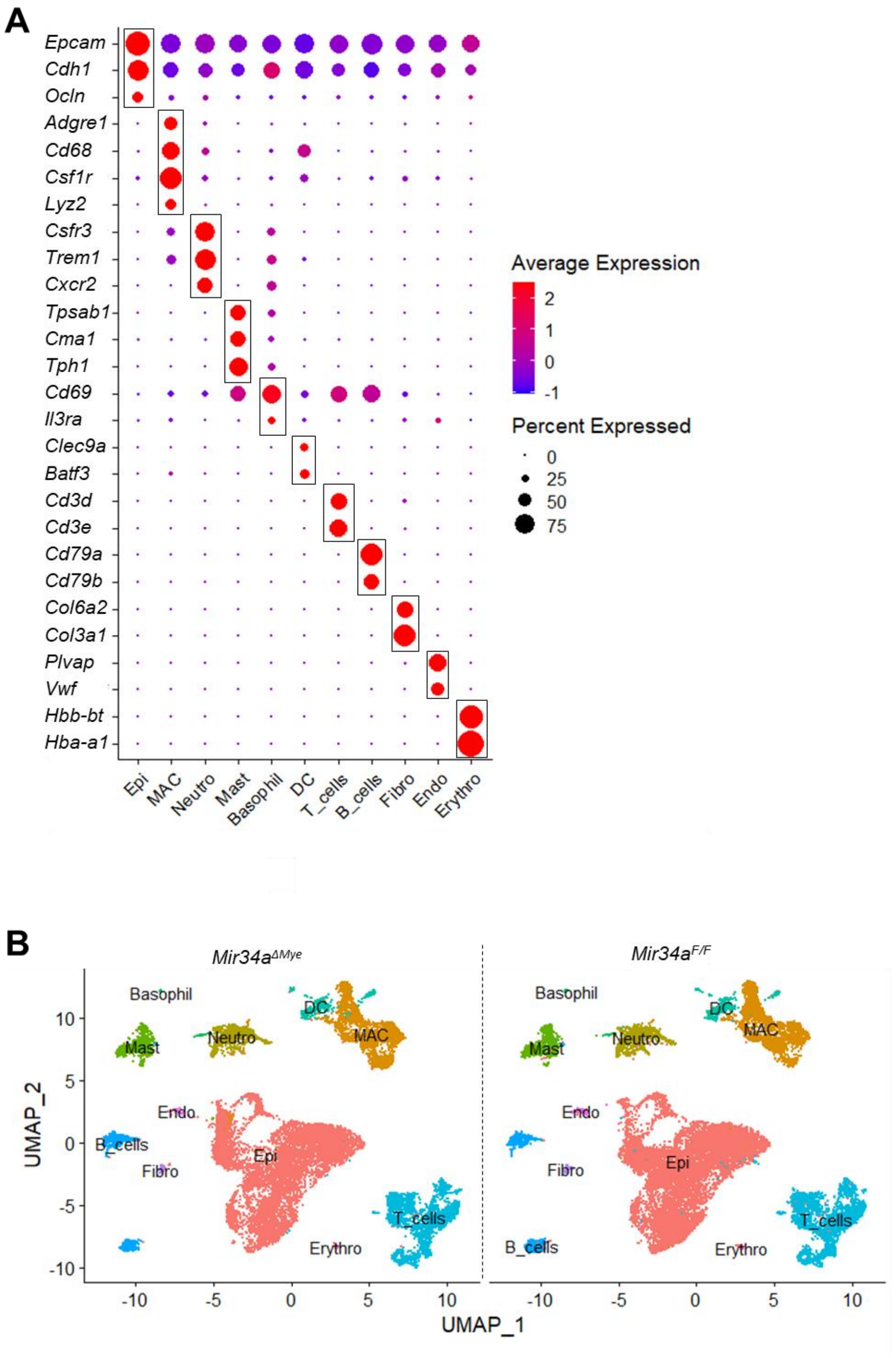

Figure S1. Related to Figure 2. **(A)** Expression of cell type marker mRNAs in the indicated cell types. **(B)** UMAP plots of the indicated cell types derived from *Mir34a*$^{\Delta Mye}$ and *Mir34a*$^{F/F}$ CACs.

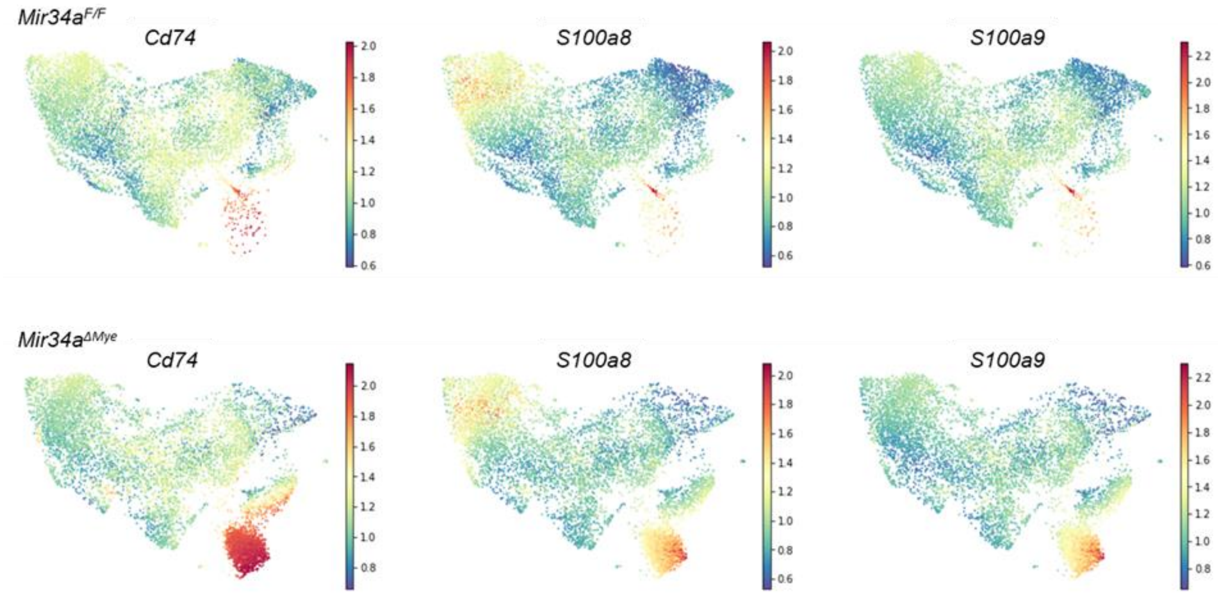

Figure S2. Related to Figure 3. UMAP plots of tumor cells pseudo-colored by the degree of expression of indicated mRNAs.

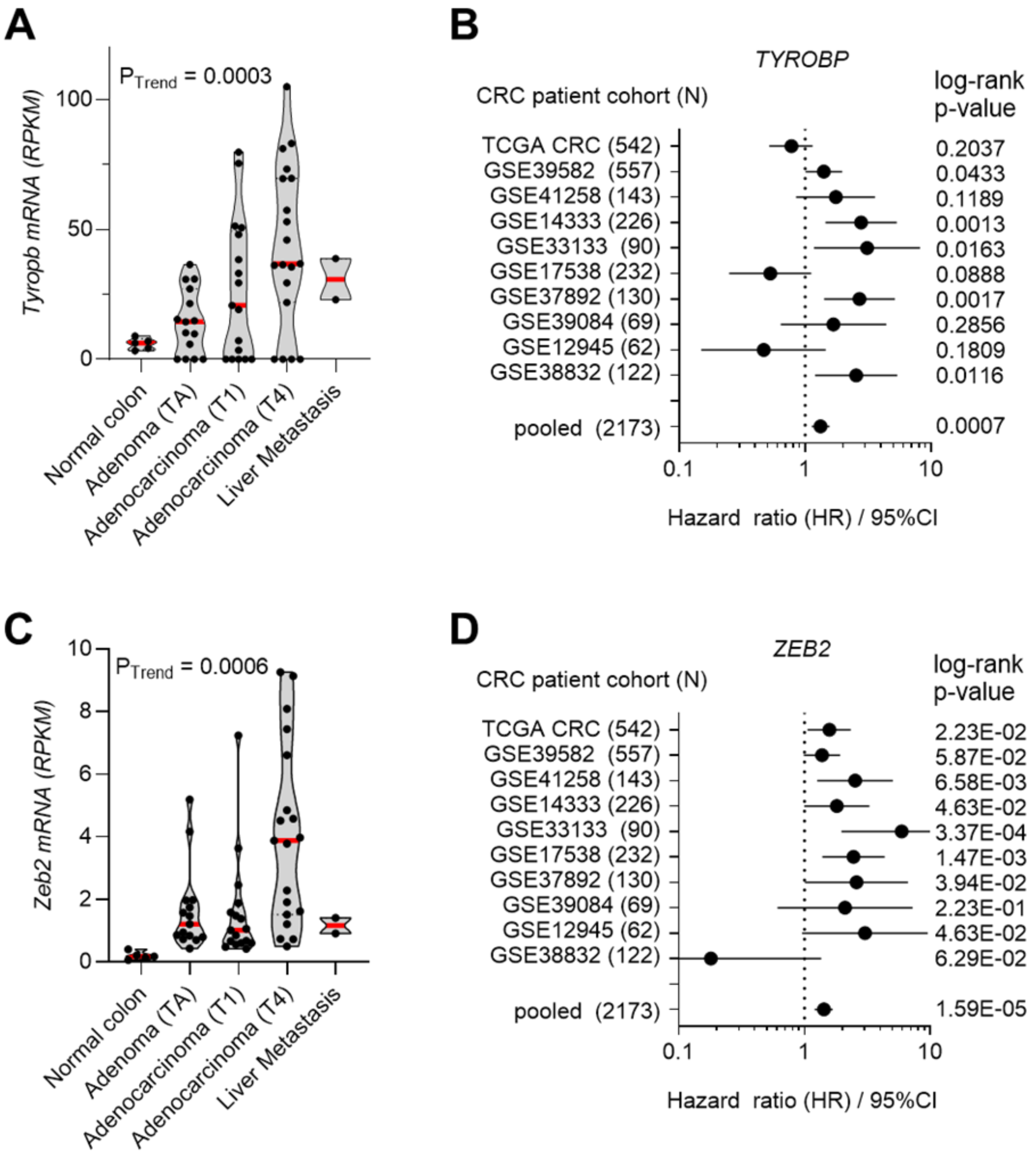

Figure S3. Related to Figure 3. **(A, C)** Expression of *Tyrobp* (A) and *Zeb2* (C) mRNAs in the *iKAP* mouse model. NC, normal colon; TA, non-invasive colon tumors; T1, low-invasive colon tumors; T4, high-invasive colon tumors; LM, liver metastases. Significance was calculated using ANOVA with post-test for linear trend from NC to LM. **(B, D)** Relapse free survival of CRC patients from indicated cohorts stratified by the expression of *TYROBP* (B) and *ZEB2* (D) mRNAs. Dots represent Hazard ratios and horizontal lines show 95% CI. P-values were calculated using the log-rank method.

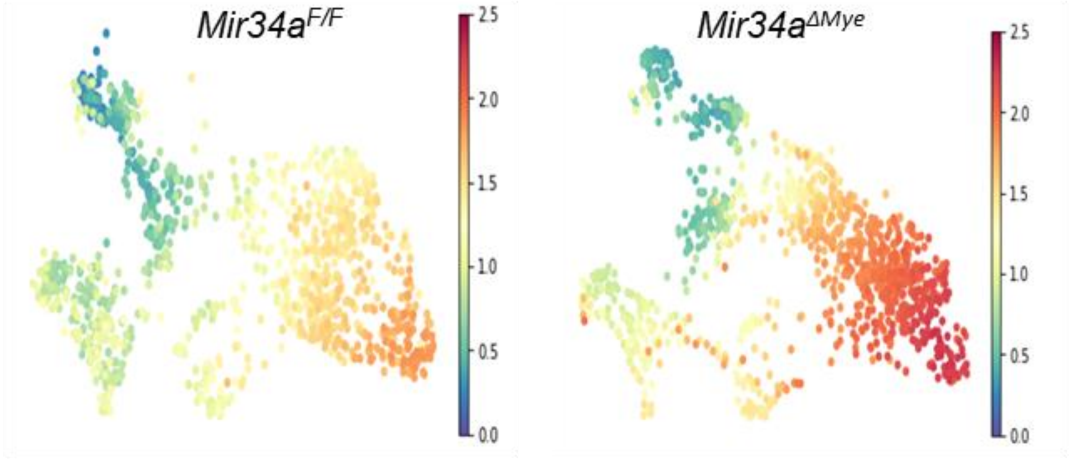

Figure S4. Related to Figure 4. UMAP plots of neutrophils pseudo-colored by the degree of expression of *Mmp9* mRNA.

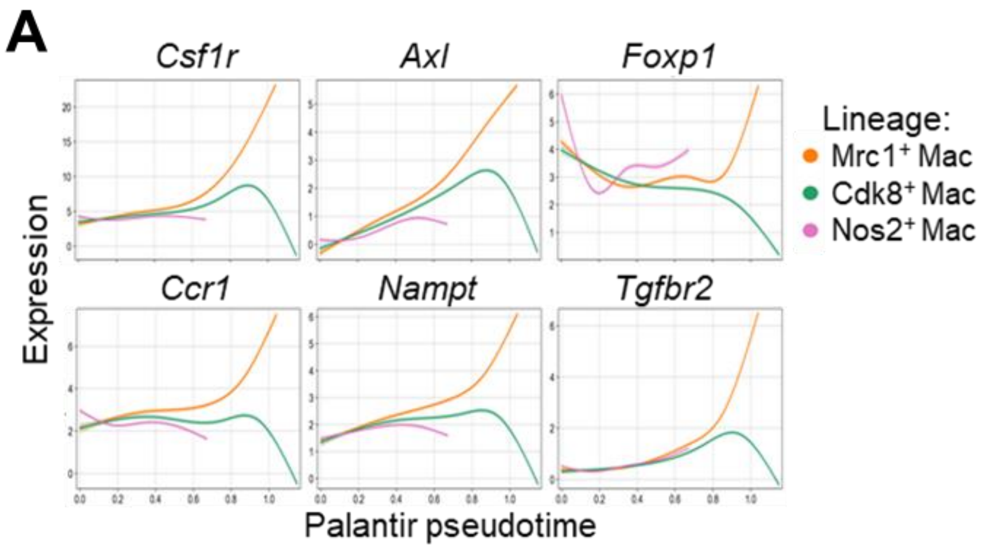

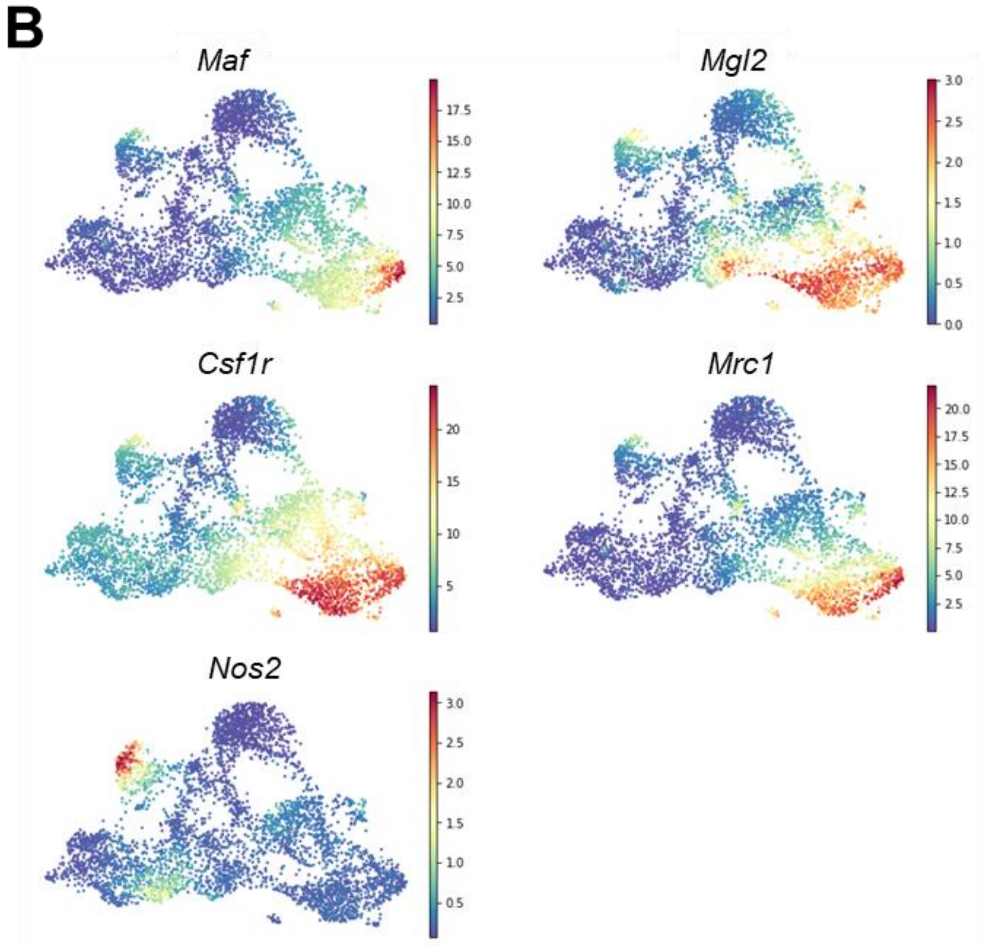

Figure S5. Related to Figure 5. **(A)** Expression trends of the indicated miR-34a target mRNAs in different macrophages lineages as a function of pseudotime. **(B)** UMAP plots of macrophages pseudo-colored by the degree of expression of the indicated mRNAs.

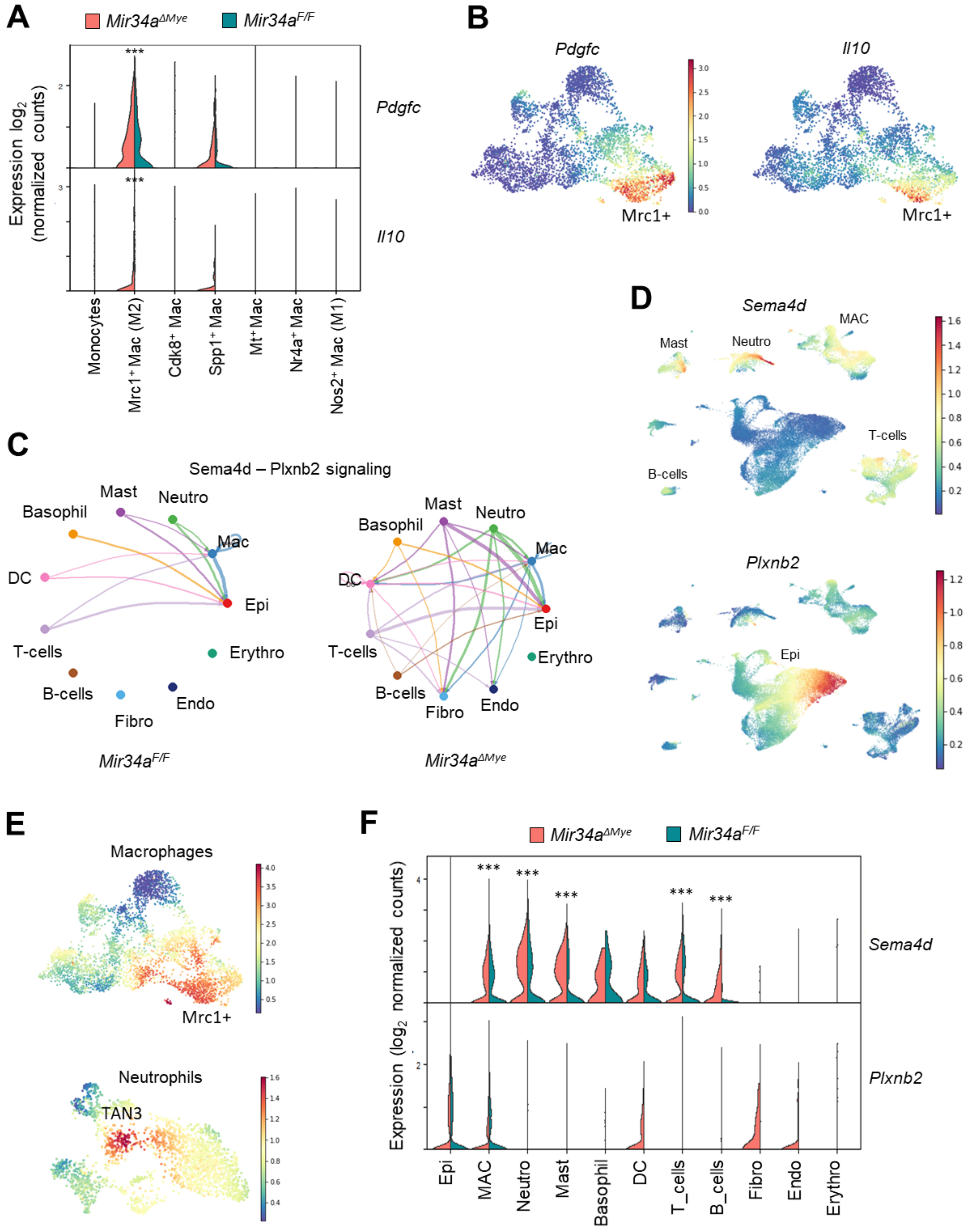

Figure S6. Related to Figure 5. **(A)** Expression of *Pdgfc* and *Il10* mRNA in indicated macrophage subtypes in *Mir34a*$^{\Delta Mye}$ and *Mir34a*$^{F/F}$ CACs. **(B)** UMAP plots of macrophages pseudo-colored by the degree of expression of the indicated mRNAs. **(C)** Sema4d – Plxnb2 signaling networks between indicated cell types in *Mir34a*$^{\Delta Mye}$ and *Mir34a*$^{F/F}$ CACs. The edge width represents the communication probability. **(D)** UMAP plots pseudo-colored by the degree of expression of the indicated mRNAs in

indicated cell types. **(E)** UMAP plots of macrophages and neutrophils pseudo-colored by the degree of expression of *Sema4d* mRNA. **(F)** Expression of *Sema4d* and *Plxnb2* mRNAs in indicated cell types in $Mir34a^{\Delta Mye}$ and $Mir34a^{F/F}$ CACs.

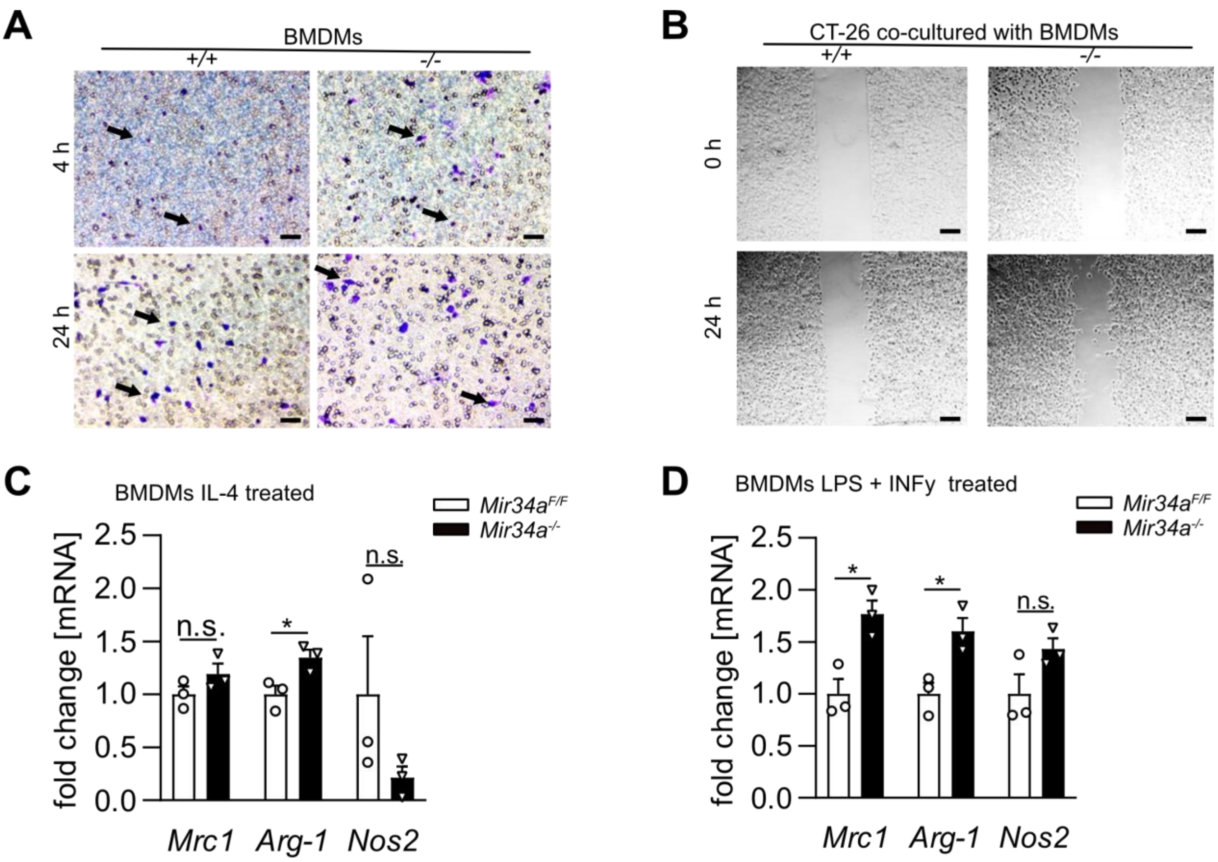

Figure S7. Related to Figure 6. **(A)** Modified Boyden chamber assay was performed in triplicate inserts. Crystal violet staining of BMDMs, which migrated through 8 µm pores. Arrows indicate BMDMs. Scale bars represent 20 µm. **(B)** Wound closure of CT-26 cells co-cultured with *Mir34a*-proficient or *Mir34a*-deficient BMDMs was performed in triplicate inserts for 24 hours. Scale bars represent 50 µm. **(C)** Expression of *Mrc1*, *Arg-1* and *Nos2* in *Mir34a*-proficient and *Mir34*-deficient BMDMs after treatment with IL-4 for 4 hours. **(D)** Expression of *Mrc1*, *Arg-1* and *Nos2* in *Mir34a*-proficient and *Mir34a*-deficient BMDMs after treatment with LPS + INFy for 4 hours. Students t-test was used to determine significance. Values represent the mean ±SEM. P-values <0.05 were regarded as statistically significant (p<0.05 *; p<0.01**; p<0.001*** and p<0.0001****).

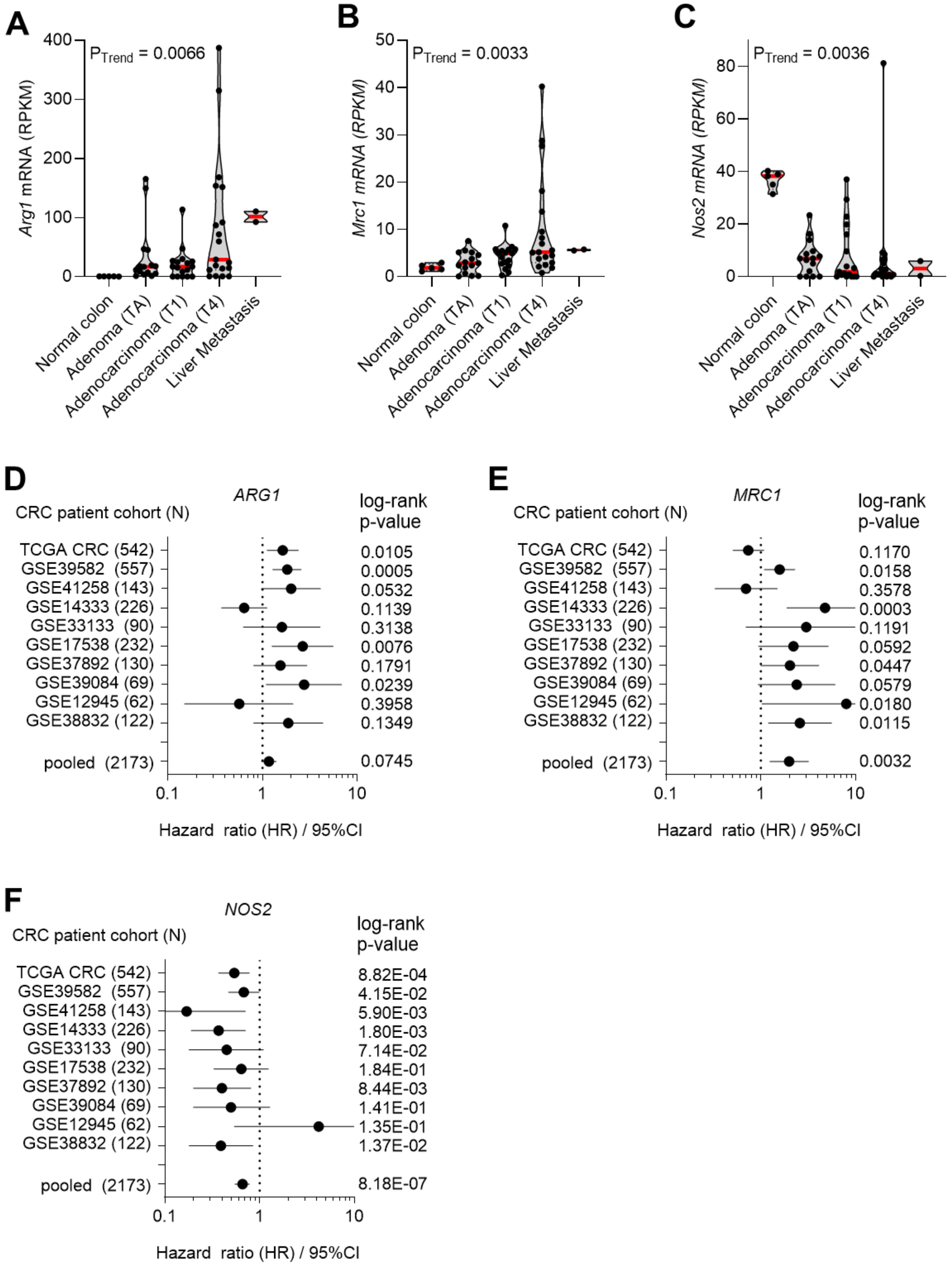

Figure S8. Related to Figure 6. **(A-C)** Expression of the indicated mRNAs in the *iKAP* mouse model. NC, normal colon; TA, non-invasive colon tumors; T1, low-invasive colon tumors; T4, highly-invasive colon tumors; LM, liver metastases. Significance was calculated using ANOVA with post-test for linear trend from NC to LM. **(D-F)** Relapse free survival of CRC patients from indicated cohorts stratified by the expression of *ARG1* (D), *MRC1* (E), and *NOS2* (F). Dots represent Hazard ratios and horizontal lines show 95% CI. P-values were calculated using the log-rank method.

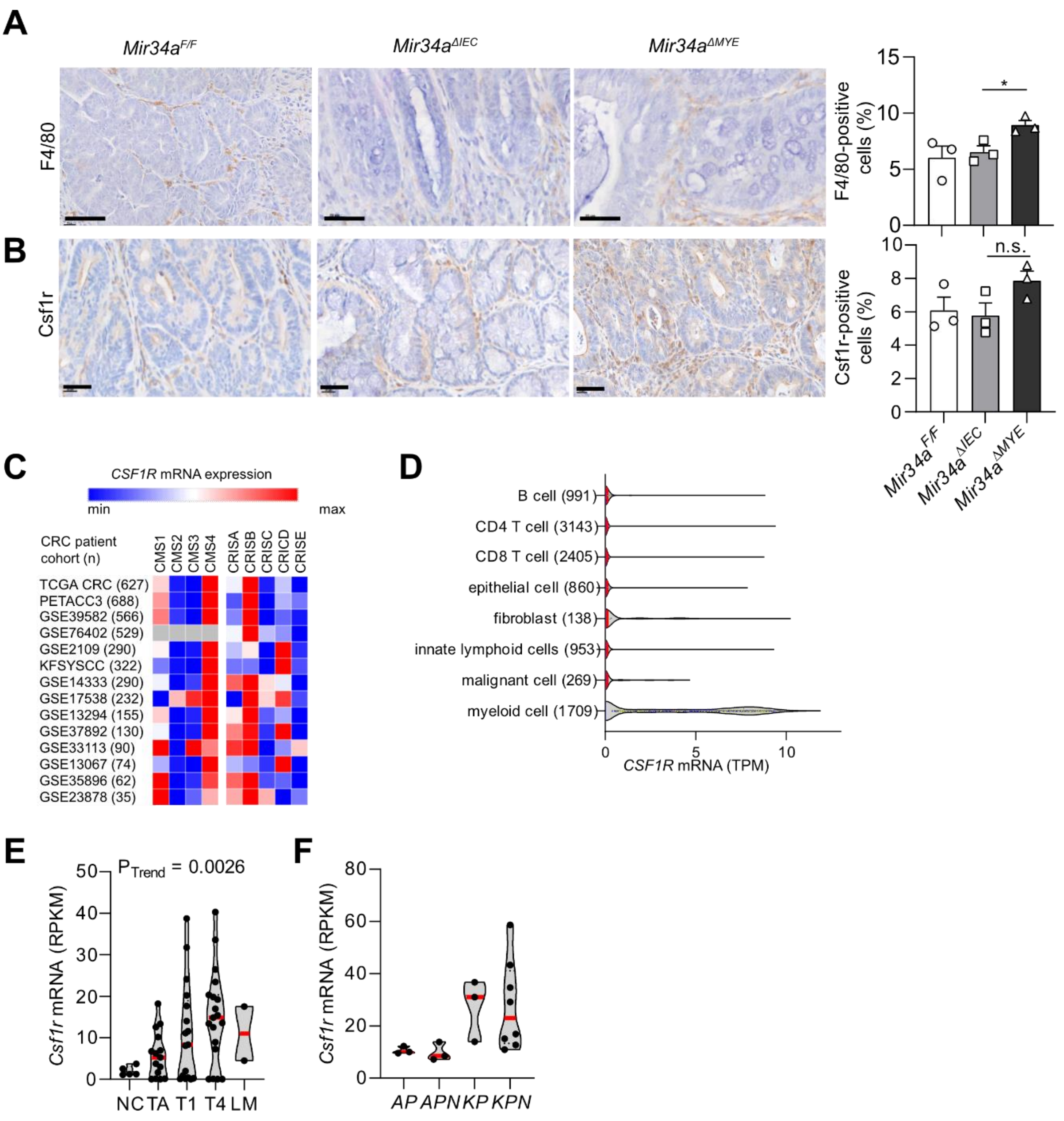

Figure S9. Related to Figure 7. **(A)** Immunohistochemical analysis of F4/80 and **(B)** Csf1r in CACs of *Mir34a$^{F/F}$*, *Mir34a$^{\Delta IEC}$* and *Mir34a$^{\Delta MYE}$* mice (n=3 mice per genotype). Scale bars represent 40 µm. Students t-test was used to determine significance. Values represent the mean ±SEM. **(C)** Expression of *CSF1R* mRNA in the indicated consensus molecular subtypes of CRC (CMS) and CRC intrinsic subtypes (CRIS) in the indicated CRC patient cohorts. **(D)** Expression of *CSF1R* mRNA in the indicated cell types within colon tumors (data is from the GSE146771 single cell RNA-Seq analysis). **(E)** *Csf1r* expression in the *iKAP* mouse model. NC, normal colon; TA, non-invasive colon tumors; T1, low-invasive colon tumors; T4, high-invasive colon tumors; LM, liver metastases. Significance was calculated using ANOVA with post-test for linear trend from NC to LM. **(F)** *Csf1r* expression in colon tumors from indicated mouse models. AP, mutant APC and p53; APN, mutant APC, p53, and activated NOTCH1; KP, mutant KRAS and p53; KPN, mutant KRAS, p53, and activated NOTCH1. P-values <0.05 were regarded as statistically significant (p<0.05 *; p<0.01**; p<0.001*** and p<0.0001****).

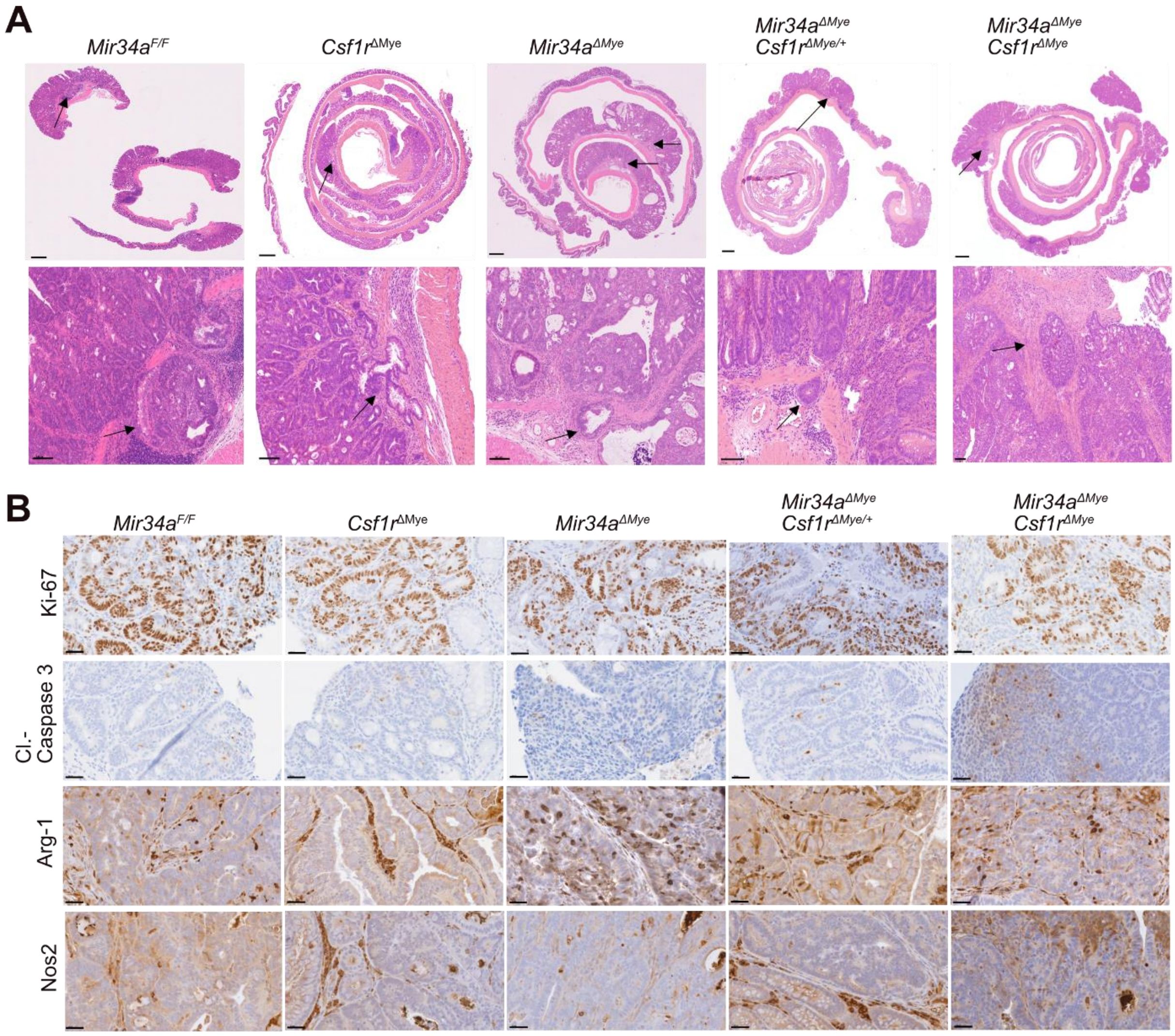

Figure S10. Related to Figure 7. **(A)** H&E stained colon sections of mice with the indicated genotypes. Arrows indicate invasive areas. Lower panels show magnified areas Scale bars represent 800 µm in the upper panel and 100 µm in the lower panel. **(B)** Immunohistochemical detection of Ki-67, cleaved-Caspase 3, Arginase-1 and Nos2 protein expression in CACs from mice with the indicated genotypes. Scale bars represent 40 µm.

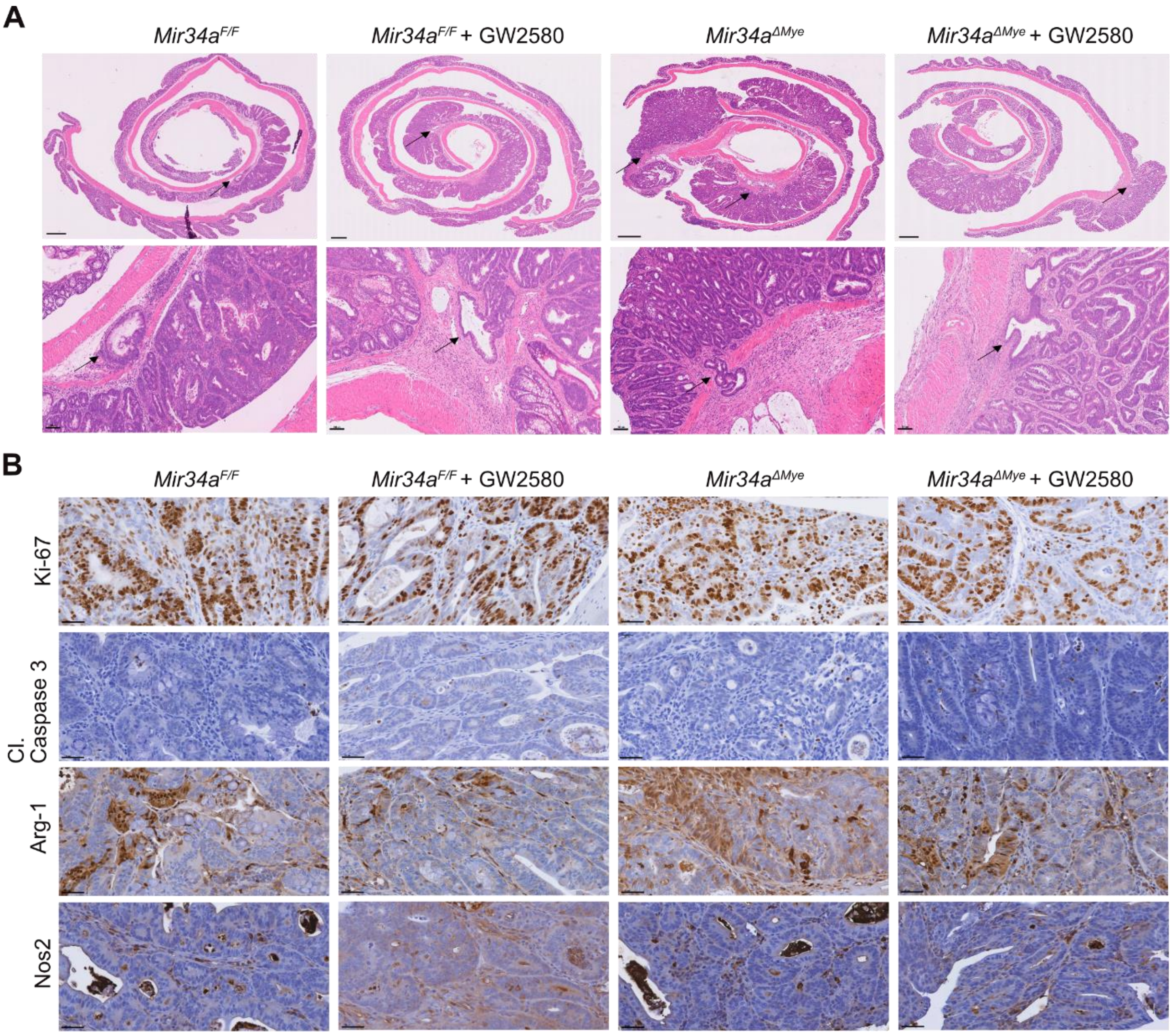

Figure S11. Related to Figure 8. **(A)** H&E stained colon sections of mice with the indicated genotypes. Arrows indicate invasive areas. Lower panels show magnified areas Scale bars represent 800 µm in the upper panel and 100 µm in the lower panel**.** **(B)** Immunohistochemistry of Ki-67, cleaved-Caspase 3, Arginase-1 and Nos2 of mice with the indicated genotypes. Scale bars represent 40 µm.

Supplemental References

1. Pfaffl MW. A new mathematical model for relative quantification in real-time RT-PCR. Nucleic Acids Res. 2001;29(9):e45.

2. Moolenbeek C, Ruitenberg EJ. The "Swiss roll": a simple technique for histological studies of the rodent intestine. Lab Anim. 1981;15(1):57-9.

Uncropped Western blot membranes:

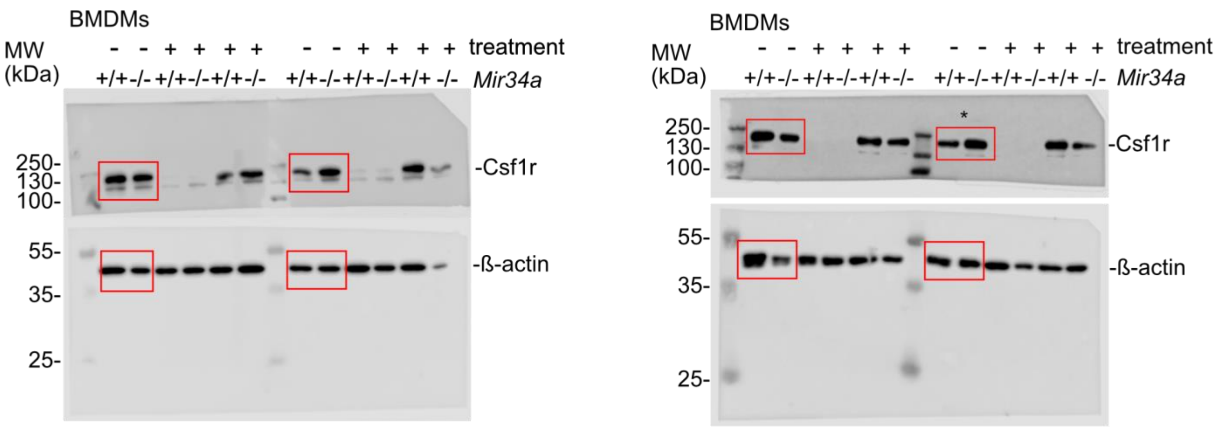

Uncropped membranes of the Western blot analysis shown in Figure 7C. Red boxes show *Mir34a*-proficient (left lane) and *Mir34a*-deficient (right lane) untreated BMDMs. * indicating membrane areas shown in Figure 7C.
